# Basic Human Body Dimensions Relate to Alcohol Dependence and Predict Hospital Readmission

**DOI:** 10.3390/jcm8122076

**Published:** 2019-11-27

**Authors:** Bernd Lenz, Martin G. Köllner, Christiane Mühle, Christian Weinland, Johannes Kornhuber

**Affiliations:** 1 Department of Psychiatry and Psychotherapy, Friedrich-Alexander University Erlangen-Nürnberg (FAU), Schwabachanlage 6, D-91054 Erlangen, Germany; christiane.muehle@uk-erlangen.de (C.M.); christian.weinland@uk-erlangen.de (C.W.); johannes.kornhuber@uk-erlangen.de (J.K.); 2 Human Motivation and Affective Neuroscience Lab, Department of Psychology, Institute of Psychology, Friedrich-Alexander University Erlangen-Nürnberg (FAU), Nägelsbachstraße 49 b, D-91052 Erlangen, Germany; martin.koellner@fau.de

**Keywords:** Alcohol Dependence, Relapse Prediction, Body Measurements, Body Dimensions, Organizational Sex Hormone Effects, Anthropometry, Pubertal Hormones

## Abstract

Alcohol dependence is a severe mental illness and there is a need for more effective preventive and therapeutic strategies. Translational research suggests that intrauterine sex hormone exposure modulates the risk and course of alcohol dependence during adulthood. During development, sex hormones permanently shape sexually dimorphic body dimensions. Thus, these dimensions may provide insight into sex hormone organization. Here, we compared body measurements (absolute, relative to, and residualized on height) between 200 alcohol-dependent in-patients and 240 age-matched healthy control subjects and investigated how these measurements associate with the patients’ prospective 12- and 24-month outcome. The results show that alcohol dependence is related to lower absolute, relative, and residualized body measurements for height and weight, head circumference, bitragion head arc, lip-chin distance, hip, thigh, and calf circumference, and foot length and breadth. In male alcohol-dependent in-patients, higher risk, shorter latency, and more alcohol-related readmissions were predicted by higher absolute, relative, and residualized thigh and calf circumferences. The second-to-fourth finger length ratio, a putative proxy for prenatal sex hormone organization, was not convincingly correlated with the body dimensions, suggesting that the results represent pubertal (or later) effects. The study’s findings have implications for further research. The body measurements’ high accessibility may facilitate the future transition into clinical settings.

## 1. Introduction

The World Health Organization (WHO) estimated that the harmful use of alcohol accounted for 5.1% of all deaths and 5.3% of all disability-adjusted life years during 2016. Worldwide, the highest levels of alcohol consumption per capita were found within the European region [1]. To develop preventive and individualized treatment strategies, we need to improve the knowledge of the mechanisms that underlie alcohol dependence. Easily accessible and clinically relevant biomarkers for both alcohol dependence per se and its prospective course are required.

In 2016, males in all WHO regions were more likely to be current alcohol drinkers and had heavy drinking sessions more often than females. In the European Region, 14.8% of males and 3.5% of females suffered from alcohol use disorder. Globally, 2.3 million male deaths compared to 0.7 million female deaths have been attributed to alcohol [1]. Alcohol dependence often runs a chronic course, with higher 12-month alcohol-related readmission rates following withdrawal in males (57.5%) compared to females (41.4%) [2]. Men are also known to have higher striatal dopamine release after oral alcohol intake than women [3]. These sex differences suggest that sex hormone activities are involved in alcohol dependence. This assumption is supported by studies that associate alcohol dependence and its related phenotypes with genes that are involved in sex hormone signaling, biosynthesis, and degradation (estrogen receptor 1 [4,5], androgen receptor [6,7], aromatase [8], 5α-reductases [9,10]). However, to our knowledge, the cited genetic links still lack replication and only one association originates from a genome-wide association study [4].

Animal and human studies indicate that during key periods such as the prenatal window and puberty, sex hormones organize the development of the brain with lasting behavioral effects and, in parallel, shape the body with lifelong consequences [11,12,13]. Hence, permanent sex dimorphisms in many human body dimensions are established during certain developmental episodes and thereafter remain stable or at least do not severely change throughout life. Thus, differences in body dimensions between males and females may be used to investigate the associations of organizing prenatal, pubertal, and later sex hormone exposures with psychiatric disorders, which include alcohol dependence.

The second-to-fourth finger length ratio (2D:4D) has been proposed as a proxy for the intrauterine sex hormone exposure, with lower values indicating prenatal hyperandrogenization [14,15] (for a critical review see [16,17]). Lower 2D:4D has been associated with a whole range of impairments such as behavioral problems in childhood [18], video game addiction [19], problematic and pathological internet use during adolescence [20], suicide in adulthood [21,22], and reduced life expectancy [23,24,25].

Lower 2D:4D has been reported in males and females that have high alcohol consumption defined as “daily” or “daily+” in a large BBC internet study [26] and as “at least two problems in the CAGE test and alcohol consumption of at least a few times each week” in a young university student sample [27]. Our first investigation on the 2D:4D-alcohol dependence relationship revealed lower 2D:4D in both males and females that were diagnosed with alcohol dependence [28]. Three additional studies on this topic have since been published [25,29,30]. In support of the initial investigation, our recent meta-analysis confirmed lower 2D:4D in alcohol-dependent males with a medium effect size (Hedge’s g = −0.552) [31]. Lower 2D:4D has also been shown to predict more alcohol-related 12-month hospital readmissions in alcohol-dependent in-patients [29]. These results suggest that higher androgen load during early intrauterine development increases the risk of alcohol dependence in adulthood and also predicts a worse outcome in affected in-patients. This early sex hormone activity model for alcohol dependence [32] is in agreement with a rodent study that demonstrated that prenatal androgen receptor inhibition reduces alcohol consumption in adult male mice, and that prenatal androgen receptor stimulation increases alcohol consumption in adult female mice [12]. Further support is provided by the fact that the opioid receptor mu 1 may interact with prenatal androgen exposure [12] and 2D:4D [33] to influence alcohol consumption during adulthood.

Another method to investigate prenatal influences of androgens on adult behaviors is the twin testosterone transfer model (TTT). The TTT is based on the assumption that compared to a female twin, a male twin increases the prenatal androgen load of his co-twin in the shared intrauterine environment [34,35]. A study using a discovery sample and an independent replication sample has shown that in males, a male co-twin reduces the risk for alcohol dependence during adulthood; in this study, there was no significant effect of the co-twin’s sex on risk of alcohol dependence in females [36]. Another study exclusively on females showed more lifetime alcohol use disorder symptoms in women with a male co-twin than in women with a female co-twin [37]. The inconsistent results in females may indicate false positive findings. By contrast, the consistent observations in males suggest that higher androgen load during early intrauterine development might decrease the risk of alcohol dependence in adulthood, which is in line with the early sex hormone activity model of alcohol dependence. However, it also implies a protective effect of higher prenatal androgen exposure in males, which may seem contradictory to the 2D:4D results described above. 2D:4D and the TTT may provide insight regarding the different prenatal developmental windows. 2D:4D sex dimorphisms are thought to originate during early pregnancy (i.e., before gestational week 9 [38] and 14 [39]). Thus, 2D:4D is believed to represent a marker of the first trimester. Because androgen levels in male fetuses peak between gestational weeks 12 and 18, the TTT may be used to investigate phenotypes that are established during the second trimester [35]. However, other explanations might also account for the inconsistent findings of studies using 2D:4D and those based on the TTT. Opposite- and same-sex human twins may differ in postnatal social factors (e.g., peer group characteristics) which also influence the risk to develop alcohol dependence later in life. Such bias may entail misinterpretation of results based on the TTT. Additionally, it cannot be ruled out that some of the above cited investigations have produced false positive findings.

In summary, a direct preclinical study and human investigations using the indirect biomarker 2D:4D and the TTT suggest that sex hormone exposure during development modulates the risk of alcohol dependence during adulthood. The organizational effects of sex hormones may depend on the developmental window during which they occur. Although an initial clinical study is being conducted to transfer this knowledge into prevention techniques [40], the field is subject to several limitations that include weaknesses related to 2D:4D and the TTT. Today, we are far from using such knowledge to improve the treatment of alcohol-dependent patients.

Moreover, little is known about the organizational effects of sex hormone exposures during puberty (and later) on alcohol dependence during adulthood. Only recently, the novel research area on sex hormone organization during the pubertal window has evolved [13]. Pubertal sex hormones shape sexual dimorphisms in physical traits and these might, therefore, be used to enlighten the associations between behavioral traits and pubertal sex hormone exposures; e.g., meta-analyses have demonstrated that the facial width-to-height ratio, which is subject to a sexual dimorphism appearing during puberty [41], is related to aggression and threat behavior among males and to dominance behavior in both sexes [42,43].

As far as we know, the association of easily measurable and thus highly accessible sexually dimorphic body dimensions with alcohol dependence and the prospective course of affected in-patients has not been systematically investigated. 

### Aims of the Study

Sex dimorphisms in body dimensions can provide initial insights into the organizational effects of sex hormones on the brain with clinically relevant behavioral outcomes. Thus, we analyzed here the differences in 15 absolute body measurements, 13 body measurements divided by height (= relative), and 13 body measurements residualized on height between alcohol-dependent in-patients and healthy control subjects of both sexes. We also investigated their relationships to the patients’ prospective 12- and 24-month outcome, following alcohol withdrawal treatment, in an effort to identify parameters that will be helpful to determine the individual prognosis. Finally, we explored the associations between absolute, relative, and residualized body measurements with 2D:4D, the age of onset of regular alcohol drinking, the age of the first in-patient treatment due to alcohol problems, total lifetime drinking, and daily ethanol intake.

## 2. Materials and Methods

### 2.1. Study Sample

This project was part of the bicentric, cross-sectional, and prospective Neurobiology of Alcoholism (NOAH) study, which included 200 alcohol-dependent in-patients and 240 age-matched healthy controls subjects [29]. All in-patients were diagnosed with alcohol dependence according to the tenth revision of the International Classification of Diseases (ICD-10) criteria [44] and alcohol use disorder according to the fifth edition of the Diagnostic and Statistical Manual of Mental Disorders (DSM-5) criteria [45]. During the screening procedure, alcohol-dependent in-patients were excluded due to denial of written informed consent (*n* = 379), a mental comorbidity, such as primary depression (i.e., depression was present before the alcohol dependence), anxiety disorder, schizophrenia, substance use disorder other than alcohol or nicotine, posttraumatic stress disorder, or eating disorder (*n* = 194), a severe somatic disorder (*n* = 90), abstinence longer than 72 hours prior to inclusion (*n* = 88), or other reasons (*n* = 37). Healthy control subjects were hired with online advertisement and distribution of flyers. After a multistep screening procedure of 1215 interested individuals, 240 healthy control subjects were enrolled. The exclusion criteria of the control group included severe somatic disorders, prior medical treatment due to alcohol dependence, ≥2 affirmed CAGE questions [46], prior psychiatric in-patient treatment, psychiatric or psychotherapeutic outpatient treatment during the past 10 years, and an Alcohol Use Disorders Identification Test (German version [47]) score > 12.

The NOAH study design included two study-visits for alcohol-dependent in-patients and one study-visit for healthy control subjects. For in-patients, the first study-visit took place during early abstinence (i.e., 24–72 h of abstinence according to the in-patients’ self-reports of their last alcohol intake prior to study inclusion); 81.5% of the in-patients attended the second direct study-visit, which took place during the median 5th day post-inclusion (interquartile range (IQR) 3–6). The subjects were recruited at the Psychiatrische und Psychotherapeutische Klinik of the Universitätsklinikum Erlangen and at the Klinik für Psychiatrie, Sucht, Psychotherapie und Psychosomatik of the Klinikum am Europakanal, Germany. Post-inclusion, we followed the patients’ records for 12 and 24 months to analyze the outcome parameters: alcohol-related readmissions per se, latency (in days) to the first alcohol-related readmission, and total number of alcohol-related readmissions during the 12- and 24-month follow-up periods. For statistical analyses, days to first readmission were set to 365 (12-month follow-up) and 730 days (24-month follow-up) in patients without any recorded alcohol-related readmission during the observation period. Clinically experienced psychiatrists and well-trained and regularly supervised doctoral students (from our study team) conducted the semi-structured interviews. The interviews at study enrolment included the age of onset of regular alcohol drinking (i.e., daily over at least 7 days), the age of first in-patient treatment due to alcohol problems, the alcohol lifetime drinking history [48], and the number of previous in-patient withdrawal treatments in the group of alcohol-dependent in-patients, the Alcohol Use Disorders Identification Test (German version [47]) for the group of healthy control subjects, and smoking status for both groups. We also quantified the breath alcohol concentration in alcohol-dependent in-patients. Moreover, carbohydrate-deficient transferrin (CDT) was measured in serum samples of both groups (Central Laboratory of the University Hospital Erlangen, Germany, DIN EN ISO 15189 accredited). For previously published articles of the NOAH study, see [2,5,29,33,49,50,51,52].

### 2.2. Ethical Approval

This study was approved by the Ethics Committee of the Medical Faculty from the Friedrich-Alexander University Erlangen-Nürnberg (ID 81_12 B; April 19, 2012). All participants provided written informed consent. The study is in accordance with the ethical principles of the World Medical Association (sixth revision of the Declaration of Helsinki, Seoul 2008).

### 2.3. Basic Human Body Dimensions

We selected body height, body weight, and mandibular arc as established, sexually dimorphic features [53,54,55]. The lip-chin distance was included as an indicator for jaw height, which is a constituent part of facial masculinity [56]. We used lip-chin distance instead of full jaw height to exclude lower lip height, which is larger in females than males [57]. The waist and hip circumferences were investigated as sexually dimorphic traits that are largely determined during puberty [58]. Additional parameters with likely relationships to skeletal and/or soft-tissue growth in general were included, such as head, thigh, calf, and ankle circumference, bitragion and sagittal head arcs, foot length and breadth.

For the measurements, the participants wore only underwear and took off their shoes. Body height, body weight, head circumference, bitragion and sagittal head arcs, wrist, waist, hip, thigh, and calf circumferences, as well as foot length and breadth were quantified according to the Basic human measurements for technological design—Part 1: Body measurement definitions and landmarks (ISO 7250-1:2008); German version EN ISO 7250-1:2010. For body dimensions not included in the above list, we used the following definitions: Mandibular arc, distance between the two anguli mandibulae spanning over the point of the chin; lip-chin distance, lower lip mucocutaneous boundary to platysma mandible insertion; hip circumference, length over the widest part of the gluteal region; ankle circumference, maximum length parallel to the floor over the malleoli mediales and laterales. Body weight was measured in kg using a weighing scale and length dimensions in cm with a tape measure. All measurements were conducted once, by a non-blinded rater, in the standing position with the exception of foot length and breadth. Soles of the right and left feet were scanned in the sitting position using an HP Scanjet G4050 (HP Deutschland GmbH, Böblingen, Germany). Foot length and breadth were quantified by three independent and blinded raters with Microsoft PowerPoint (two-way random intra-class correlation coefficients (absolute agreement): mean of right and left foot length 0.996, mean of right and left foot breadth 0.990). We did not exclude hallux valgus deformations. Most of the missing values in the alcohol-dependent in-patients were due to a lower participation rate in the second study-visit, during which body measurements were conducted (if not already done during the first study visit).

### 2.4. 2D:4D

Right and left hands were scanned using an HP Scanjet G4050. The absolute index and ring finger lengths were quantified by three independent blinded raters and we analyzed the means of the right-hand and left-hand 2D:4D values, which are published (GNU Image Manipulation Program, www.gimp.org, two-way random intra-class correlation coefficient (absolute agreement) of mean of the right-hand and left-hand 2D:4D 0.986) [29].

### 2.5. Statistical Analyses

Data were analyzed using SPSS for Windows 24.0 (SPSS Inc., Chicago, IL, USA). The descriptive statistics report medians, IQR, and frequencies, which were calculated with the custom tables function of SPSS. We used the Mann–Whitney *U* test and Spearman’s correlation because some of the body measurements deviated significantly from a normal distribution, according to the Kolmogorov–Smirnov test. The χ^2^ test was employed to analyze differences in the frequency of nominal variables. Values of *P* < 0.05 for two-tailed tests were considered significant. To respect the well-known sex differences in alcohol dependence [32], we analyzed males and females separately.

Mean values of right and left-side body measurements were analyzed for wrist, thigh, calf, and ankle circumferences as well as foot length and breadth. We tested for associations of absolute body measurements, body measurements divided by height, and body measurements residualized on height with alcohol dependence and outcome. Body measurements were divided by height to obtain a relative measure in analogy to those frequently used in other fields of marker research (e.g., 2D:4D, facial width-to-height ratio). Moreover, the absolute values of body measurements were residualized for height to determine possible sex differences in and the predictive value of each parameter above and beyond general differences in body height. Linear regression, using the whole study population, was applied to residualize each single body measurement (dependent variable) on body height (predictor). The obtained residuals were interpreted as body measurement values that are independent of general body height. Residuals were z-standardized.

The field of organizational sex hormone effects in alcohol dependence is early in development. Hence, we tested a high number of predictors. In total, we predefined the following 656 statistical tests:

- 82 group comparisons “alcohol-dependent in-patients versus healthy control subjects”: (15 absolute body measurements + 13 body measurements divided by height + 13 body measurements residualized on height) × 2 sexes
- 164 group comparisons “readmission yes versus no”: (15 absolute body measurements + 13 body measurements divided by height + 13 body measurements residualized on height) × 2 sexes × 2 follow-up periods (12-month, 24-month)
- 328 correlations with “outcome parameters”: (15 absolute body measurements + 13 body measurements divided by height + 13 body measurements residualized on height) × 2 sexes × 2 follow-up periods (12-month, 24-month) × 2 outcome parameters (latency, number)
- 82 group comparisons “male versus female”: (15 absolute body measurements + 13 body measurements divided by height + 13 body measurements residualized on height) × 2 groups (alcohol-dependent in-patients versus healthy control subjects)

All reported *P* values are uncorrected. To consider type 1 error risk in multiple hypothesis testing, we employed the false discovery rate (FDR) procedure using a macro for Microsoft Excel (see Appendix S1 of Pike [59], classical one-stage method including all 656 tests; critical *P* value (FDR-derived significance threshold): 0.007470; [60]). In parallel, we also employed the more conservative Bonferroni adjustment (critical *P* value: 0.05/656 = 0.000076). We chose both correction methods to differentiate between robust (FDR) and very robust (Bonferroni) results. However, we acknowledge the problem of statistical power in view of the high number of tests. The sample size has been initially chosen to demonstrate group differences in 2D:4D already published in [29]. The body measurements analyzed in this article represent secondary endpoints and were not included into the a priori sample size estimation.

Finally, we performed the following explorative analyses that were not corrected for multiple hypothesis testing:

- Correlations of absolute, relative, and residualized body measurements with 2D:4D in alcohol-dependent in-patients and healthy control subjects of both sexes.
- Correlations of absolute, relative, and residualized body measurements with the age of onset of regular alcohol drinking and the age of first in-patient treatment due to alcohol problems in alcohol-dependent in-patients of both sexes.
- Correlations of absolute, relative, and residualized body measurements with total lifetime drinking and daily ethanol intake in alcohol-dependent in-patients of both sexes.
- Inter-correlations among the body measurements within the three groups of absolute, relative, and residualized body dimensions in alcohol-dependent in-patients and healthy control subjects of both sexes.

## 3. Results

### 3.1. Sociodemographic Characteristics

Both male and female alcohol-dependent in-patients did not significantly differ from healthy control subjects with regard to age, but were more often active and ever-smokers and had higher CDT blood levels. In the group of alcohol-dependent in-patients, male sex was significantly related to a younger age of onset of regular alcohol drinking (i.e., daily over at least 7 days), higher total lifetime drinking and daily ethanol intake since onset, a higher alcohol concentration at admission, higher CDT blood levels, and worse 12- and 24-month outcomes. Moreover, we found significantly higher Alcohol Use Disorders Identification Test scores and higher CDT blood levels in male (than in female) healthy control subjects (Table 1).

### 3.2. Basic Human Body Dimensions: Alcohol-Dependent In-Patients versus Healthy Control Subjects

After FDR correction, several absolute, relative, and residualized body measurements were significantly and consistently lower in alcohol-dependent in-patients than in healthy control subjects, except for sagittal head arc divided by height, which was significantly longer in alcohol-dependent in-patients (Table 2).

Absolute Body Measurements: Alcohol dependence was related to shorter head, hip, and thigh circumference in both sexes and to lower body height and weight, bitragion head arc length, lip-chin distance, calf circumference, and foot length and breadth in males. Body Measurements Divided by Height: Alcohol dependence was associated with shorter thigh circumference in both sexes, with longer sagittal head arc length and shorter hip and calf circumference in males, and with shorter head circumference in females. Body Measurements Residualized on Height: Alcohol dependence was related to shorter hip and thigh circumferences in both sexes, to a shorter bitragion head arc, calf circumference, and foot breadth in males, and to shorter head circumference in females.

The smaller absolute, relative, and residualized hip, thigh, and calf circumferences and the smaller absolute body weight and foot breadth in male alcohol-dependent in-patients, in comparison to male healthy control subjects, remained significant after the Bonferroni correction.

### 3.3. Basic Human Body Dimensions: Prospective Alcohol-Related Readmission

After FDR correction, worse patients’ outcomes were consistently related to significantly higher absolute, relative, and residualized thigh and calf circumferences in male alcohol-dependent in-patients. We did not find any significant association of absolute, relative, or residualized body measurement with outcomes in female alcohol-dependent in-patients (Table 3, Table 4 and Table 5).

Absolute Body Measurements: Thigh circumference was linked to risk, latency, and the number of alcohol-related readmissions throughout the 12-month follow-up. Calf circumference correlated with the 12-month latency to alcohol-related readmission. Body Measurements Divided by Height: Thigh circumference was associated with risk, latency, and the number of alcohol-related readmissions for the 12-month and 24-month follow-ups. Calf circumference was linked to 12-month risk and 12-month and 24-month latency to alcohol-related readmission. Moreover, there was an association between head circumference values and number of alcohol-related readmissions during the 24-month follow-up. Body Measurements Residualized on Height: Thigh circumference was linked to risk, latency, and the number of alcohol-related readmission for the 12-month follow-up and the number of alcohol-related readmissions for the 24-month follow-up. Calf circumference related to 12-month und 24-month latency to alcohol-related readmission.

None of these effects remained significant after the Bonferroni adjustment.

### 3.4. Basic Human Body Dimensions: Sex Differences

After FDR correction, we found significantly longer absolute and residualized body measurements in males than in females, except for the shorter residualized thigh circumference in male than in female alcohol-dependent in-patients. Many relative body measurements were significantly lower in males than in females (Table 2).

Absolute Body Measurements: Male sex was related to higher body weight and height, head circumference, lengths of bitragion and sagittal head arc and mandibular arc, lip-chin distance, wrist, waist, and ankle circumference, and foot length and breadth in both groups and to a longer calf circumference in the healthy control subjects. Body Measurements Divided by Height: Male sex was linked to lower head circumference values, lengths of bitragion and sagittal head arc, a higher wrist circumference, and lower hip and thigh circumferences in both groups, to a smaller calf circumference and a longer foot length in alcohol-dependent in-patients, and to a higher waist circumference in healthy control subjects. Body Measurements Residualized on Height: Male sex was associated with longer head and wrist circumference in both groups, with a longer mandibular arc, shorter thigh circumference, and higher foot length in alcohol-dependent in-patients, and with longer lip-chin distance, waist and ankle circumference, and foot breadth in healthy control subjects.

Many of these sex differences remained significant after the Bonferroni correction.

### 3.5. Basic Human Body Dimensions: Exploratory Analysis of Correlations with 2D:4D, the Age of Onset of Regular Alcohol Drinking and First In-Patient Treatment Due to Alcohol Problems, Total Lifetime Drinking and Daily Ethanol Intake, and Inter-Correlations

The analyses revealed no convincing pattern of significant relationships between 2D:4D and absolute body measurements, body measurements divided by height, or body measurements residualized on height (no significant correlation except for seven tests with *P* > 0.039, Table A1 in Appendix A).

The age of onset of regular drinking (i.e., daily over at least 7 days) and the age of the subjects’ first in-patient treatment due to alcohol problems correlated positively and significantly with many absolute, relative, and residualized body measurements in both male and female alcohol-dependent in-patients; however, there were also significant negative correlations with the lengths of sagittal head arc and thigh circumference in male patients (Table A2 in Appendix A).

Absolute Body Measurements: In male alcohol-dependent in-patients, the age of onset of regular drinking and/or the age of first in-patient treatment due to alcohol problems correlated positively with lengths of mandibular arc, waist, hip, and ankle circumferences and negatively with sagittal head arc and thigh circumference. Body Measurements Divided by Height: The age of onset of regular drinking and/or the age of first in-patient treatment due to alcohol problems correlated positively with lengths of mandibular arc and waist and ankle circumference and negatively with sagittal head arc and thigh circumference in male alcohol-dependent in-patients and positively with wrist and ankle circumference and foot length and breadth in female alcohol-dependent in-patients. Body Measurements Residualized on Height: The age of onset of regular drinking and/or the age of first in-patient treatment due to alcohol problems correlated positively with lengths of mandibular arc and waist, hip, and ankle circumferences and negatively with sagittal head arc and thigh circumference in male alcohol-dependent in-patients and positively with bitragion head arc, wrist and ankle circumference, and foot length and breadth in female alcohol-dependent in-patients.

Total lifetime drinking and daily ethanol intake did not significantly correlate with absolute, relative, or residualized body measurements in male and female alcohol-dependent in-patients, except for a significant negative correlation between total lifetime drinking and absolute lip-chin distance in male patients and between daily ethanol intake and absolute, relative, and residualized mandibular arc length in male and female patients (*P* > 0.025) (Table A3 in Appendix A).

We found multiple significant inter-correlations among the absolute body measurements (Table A4 and Table A5 in Appendix A), the body measurements divided by height (Table A6 and Table A7 in Appendix A), and the body measurements residualized on height (Table A8 and Table A9 in Appendix A).

## 4. Discussion

As far as we know, this is the first systematic and comprehensive anthropomorphic study that determined the associations between basic human body dimensions and alcohol dependence. Separately for both sexes, we have found many shorter body measurements in alcohol-dependent in-patients than in healthy control subjects. These results are supported by two earlier studies that indicated that participants recruited from alcoholism in-patient treatment programs have lower body height, body weight, and thigh volume than controls [61,62], although it should be noted that these projects focused primarily on body composition and sex differences in alcohol consumption. Moreover, we showed here for the first time that in male alcohol-dependent in-patients, a higher thigh circumference may predict a worse 12-month outcome with a higher risk, shorter latency to the first alcohol-related readmission, and a higher number of alcohol-related readmissions.

We were able to verify many of the a priori expected sexual dimorphisms with shorter absolute and residualized on height physical dimensions in females than males. These results confirm the assumption that the body dimensions may provide insight into the organizational effects of sex hormones. Hence, the associations between sexually dimorphic body measurements and alcohol dependence observed here may support that the organizational effects of sex hormones are involved in alcohol dependence. Interestingly, the absolute, relative, and residualized body measurements did not convincingly correlate with 2D:4D in any subgroup. The independence of these factors may suggest that, rather than prenatal sex hormones, the pubertal (or later) sex hormones organize the physical dimensions analyzed in this study. Support of this idea comes from a rodent study that failed to show consistent effects of prenatal androgen receptor modulation on head size and body length [11]. It is therefore more likely that pubertal (or even later), rather than prenatal sex hormone effects, account for the differences between alcohol-dependent in-patients and healthy control subjects observed here. Moreover, the smaller body height of alcohol-dependent males in comparison to healthy control males detected here is contradictory to the expectations based on findings from the TTT. In males, a male co-twin in comparison to a female co-twin reduces both the risk for later alcohol dependence [36] and body height [63], although the effect sizes were small in both investigations. However, this entails the prediction of larger body height in alcohol-dependent males. The opposite finding in this study further corroborates that, rather than prenatal sex hormones, the pubertal (or later) sex hormones organize the physical dimensions analyzed here. It may also indicate that other factors as discussed in the following section are involved.

### 4.1. Possible Reasons for Smaller Body Dimensions in Alcohol Dependence

We detected many shorter absolute, relative, and residualized body measurements in alcohol-dependent in-patients than in healthy control subjects. Shorter measurements for most of the absolute and residualized parameters were also present in females than in males. At first glance, the shorter body measurements in the alcohol dependence group (compared to the control group), in light of the shorter values in females than in males, might appear contradictory to the higher prevalence of alcohol dependence in males than in females [1]. However, different reasons might account for these results.

Prepubertal boys show lower estradiol levels than prepubertal girls [64], which entails the later onset of puberty in males. Sex differences in bone mass, size, and strength strongly increase during puberty, with higher values in males compared to females [65,66]. Males are taller and develop longer bones than females because their growth spurts last longer before the epiphyses fuse, which results from the later rise and lower peak levels of estradiol (see [67], regarding sex differences in estrogen and epiphyseal fusing). Thus, lifelong smaller body measurements, which were observed here in alcohol dependence, may result from both a later pubertal onset and factors that reinforce estrogen signaling during puberty that may prematurely terminate the growth of the body. With regard to pubertal onset, we have already shown that male alcohol-dependent in-patients and male binge drinkers report a later age at puberty onset than control males [24,29], which may result in shorter body measurements. In addition, we hypothesize a premature termination of pubertal growth in the group of alcohol-dependent in-patients. Here, we found an overlap between the age of onset of regular alcohol drinking and puberty (the age of onset of regular alcohol drinking in years: minimum to 25% percentile, males 14–19, females 14–23) and 17.4% of males (16 out of 92) and 4.5% of females (3 out of 67) stated that they had started drinking alcohol regularly before turning 18 years old. Phytoestrogens are found in many alcoholic beverages. They are produced from plants that contain estrogen-like substances [68]. In addition, low dose alcohol consumption has been shown to increase testosterone levels in both sexes [69,70] and testosterone is converted to estradiol by aromatase. We speculate that the early onset of regular alcohol drinking in the groups studied here, in male, and to a lesser extent, female alcohol-dependent in-patients, may have led to a premature epiphyseal fusing via phytoestrogens and testosterone to estradiol conversion (see [67], regarding sex differences in estrogen and epiphyseal fusing, [71]). We provide some support for this model in that the age of onset of regular drinking (i.e., daily over at least 7 days) and the age of first in-patient treatment due to alcohol problems were found to correlate positively with many absolute, relative, and residualized body measurements in both male and female alcohol-dependent in-patients. However, we acknowledge that the age of onset of regular drinking was assessed retrospectively and that the correlations do not allow for causal conclusions. In a nutshell, the smaller body dimensions in alcohol dependence observed here might be explained by a shorter pubertal growth period with later ages of pubertal onset and a premature end of bone growth. Additionally, the data reported here indicate that earlier ages at the onset of regular alcohol drinking (i.e., daily over at least 7 days) in males (more than in females) may account for the stronger group differences in body measurements between males and females that were observed in this study.

Moreover, additive genetic effects explain 40% of pubertal timing in boys and 46% in girls [72], and genes involved in sex hormone signaling have previously been associated with alcohol dependence and craving for alcohol [4,5,6,8,9,10]. Hence, shared genetic variance of pubertal timing and addictive behaviors might explain why absolute, relative, and residualized body dimensions are smaller in alcohol-dependent in-patients in the analyzed cohort.

Fetal alcohol spectrum disorder (FASD) provides another explanation for the many lower values of absolute, relative, and residualized body measurements in alcohol-dependent in-patients compared to the healthy control subjects in this study. A meta-analysis reported a high prevalence of pooled alcohol or drug dependence in 54.5% of the subjects diagnosed with FASD [73]. Growth restriction including height and head circumference is a typical phenomenon in subjects with FASD, which is also present in adulthood [74]. Thus, a higher percentage of prenatal alcohol exposure in the in-patients’ cohort might also account for the smaller absolute, relative, and residualized body dimensions. We have not investigated FASD in this project and future research is necessary to further test this hypothesis. However, a recent register-based study estimated a rate of FASD of 177 children per 10,000 live births [75]. Although this study might underestimate the prevalence of FASD, it is unlikely that FASD explains the observed group differences completely.

Unhealthy nutrition and poor physical activity are frequent consequences of alcohol dependence and influence body composition, particularly the muscle and fat tissues. The smaller body dimensions, including body weight and hip, thigh, and calf circumferences in the in-patients’ groups, may represent direct sequelae of alcohol dependence. Surprisingly, total lifetime drinking and daily ethanol intake in the cohort of male and female in-patients did not convincingly correlate with absolute, relative, or residualized body measurements (except for some correlations with lip-chin distance and mandibular arc). This suggests that the individual alcohol intake over time does not largely account for the group differences in body measurements and their associations with prospective outcomes. However, the lack of a clear correlation between lifetime drinking history and body measurements might be specific to the investigated cohort, because the patients were exclusively recruited in psychiatric hospitals and we did not include in-patients with severe somatic disorders. Furthermore, alcohol-dependent in-patients in this study are more likely than healthy control subjects to identify as active (males: 78% versus 22%, females: 77% versus 19%) or ever-smoker (males: 92% versus 59%, females: 85% versus 48%). Both lifetime and current smoking might influence body dimensions and contribute to the effects observed here.

In summary, the smaller body dimensions in alcohol dependence may be due to a later age of pubertal onset, a premature end of bone growth due to the effects of alcohol, such as phytoestrogens and impaired sex hormone signaling, an increased rate of FASD, and unhealthy life-style factors that include nutrition, physical activity, and smoking behavior, which are related to alcohol dependence.

### 4.2. Thigh Circumference: Alcohol Dependence and Prospective Outcome

In this study, we found shorter thigh circumference in alcohol-dependent in-patients than in healthy control subjects. Moreover, longer thigh circumference predicted a worse prospective outcome in male alcohol-dependent patients. The latter associations with outcome did not remain significant after Bonferroni correction; although they did remain significant after FDR correction, and are thus considered robust, these results should be interpreted with caution.

In the same cohort that was analyzed here, we previously showed a lower body mass index in alcohol-dependent in-patients than in healthy control subjects [29] and an association between higher body mass index and a worse outcome in the patients’ group [52]. This parallels the pattern demonstrated here for thigh circumference. Thigh circumference mirrors a combination of both bone and soft tissue, which is a similarity to the body mass index. The smaller thigh circumference (in line with the lower body mass index) in alcohol-dependent in-patients compared to the healthy control subjects might be a consequence of malnutrition and/or inactivation. Because of a strong positive correlation between body mass index and thigh circumference (males: alcohol-dependent in-patients, *n* = 105, ρ = 0.833, *P* < 0.001, healthy control subjects, *n* = 133, ρ = 0.666, *P* < 0.001; females: alcohol-dependent in-patients, *n* = 75, ρ = 0.730, *P* < 0.001, healthy control subjects, *n* = 105, ρ = 0.807, *P* < 0.001), the relationship between higher thigh circumference and alcohol-related readmissions in male alcohol-dependent in-patients observed here might be explained by potential mediators of the association between higher body mass index and alcohol relapse, which we elaborated on in [52], such as *N*-acetylaspartate, appetite-regulating peptides, neuroticism, impulsivity, and temporal discounting. Both increased relapse risk and higher body mass index have been associated with low thalamic *N*-acetylaspartate [76,77] and neuroticism [78,79,80]. Moreover, ghrelin, which regulates energy homeostasis, influences cerebral reward pathways. Pharmacological strategies targeting ghrelin pathways are being tested in the treatment of alcohol-dependent patients [81]. Higher body mass index and obesity are associated with higher impulsivity and higher temporal discounting [82,83]. Thus, higher thigh circumference (indicating higher body mass index) may be related to higher impulsivity and temporal discounting with more difficulties to resist the urge to drink alcohol in risky situations. Future longitudinal studies are needed to investigate these hypotheses.

### 4.3. Limitations and Strengths

The present research project is subject to several limitations. We did not differentiate between bone and soft tissue [for a discussion of this topic in a pubertally organized marker, see [84]. This is especially critical for thigh circumference, which is clinically the most promising body dimension identified here. Future research, which might involve medical imaging techniques in addition to direct surface measurement, is needed to disentangle the different roles of bone and soft tissue. We wish to note that this limitation is true for a large part of marker research in general. Even the correlation between surface and x-ray-measurements of 2D:4D as an often used biomarker is comparatively low (*r* = 0.45 [85]). The related imprecision might result in an underestimation of the relationship between markers and external criteria. Moreover, we cannot conclusively determine to what extent prenatal, pubertal, and even later factors account for the observations. Here, we provided evidence in the form of associations that does not allow for drawing causal conclusions. Further studies using longitudinal designs and investigating neurobiological aspects are needed to relate the potential mechanisms underlying the development of the here quantified absolute, relative, and residualized body measurements to alcohol dependence. The field’s early stage of development resulted in many statistical tests involving absolute values, ratios, and residuals. Aiming to identify promising markers, we decided to use the less conservative FDR method to consider the type 1 error risk. Unsurprisingly, some of the significant effects disappeared after the more conservative Bonferroni adjustment. However, the results from this research project build a solid basis and reveal which body measurements merit future research to confirm that the results can be replicated and generalized. We acknowledge that the patients’ outcome data were gathered using electronic patient records; no direct resurveys have been conducted. The parameter quantified here, i.e., alcohol-related readmission, differs from alcohol relapse.

The strengths of our study include the sex- and age-balanced cohorts and the long-term follow-up of alcohol-dependent patients. We specifically addressed alcohol-related readmissions, which are substantial problems in the long-term course of alcohol dependence. We also found consistent differences between alcohol-dependent in-patients and healthy control subjects across several body dimensions and considered the absolute values, ratios, and residuals. Finally, the physical measurements are easily accessible, which facilitates the transition of the results into daily clinical routine.

## 5. Conclusions

Here, we reported consistently lower values of a line of absolute, relative, and residualized body dimensions in alcohol-dependent in-patients than in healthy control subjects across both sexes. Differences in pubertal age, the effects of alcohol drinking during puberty, such as phytoestrogens and impaired sex hormone signaling, FASD, and life-style alterations related to alcohol dependence might account for these effects. Moreover, our results may have important clinical implications, as thigh circumference has turned out to be a potential and easily measureable predictor for alcohol-related readmissions. If the results observed here can be replicated, thigh circumference might serve as a measure to assess an in-patient’s risk of prospective hospital readmissions. These findings can be utilized to establish individualized treatment strategies.

## Figures and Tables

**Table 1:** Demographic characteristics of male and female alcohol-dependent in-patients (ADP) and healthy control subjects (HCS).

	Males								Females								ADP versus HCS				Males versus Females			
	ADP (*N* = 113)				HCS (*N* = 133)				ADP (*N* = 87)				HCS (*N* = 107)				Males		Females		ADP		HCS	
	*N*	M/F	IQR		*N*	M/F	IQR		*N*	M/F	IQR		*N*	M/F	IQR		*U*/χ^2^	*P*	*U*/χ^2^	*P*	*U*/χ^2^	*P*	*U*/χ^2^	*P*
Age (years)	113	48	40	53	133	48	38	56	87	48	42	55	107	49	39	55	7369	0.794	4542	0.772	4502	0.308	6954	0.762
The age of onset of regular alcohol drinking (i.e., daily over at least 7 days) (years)	92	26	19	34		-			67	33	23	41		-							2152	**0.001**		
The age of first in-patient treatment due to alcohol problems (years)	96	36	30	47		-			77	40	32	46		-							3256	0.179		
Total lifetime drinking (kg)	88	705	364	1838		-			62	332	174	747		-							1485	**<0.001**		
Daily ethanol intake (g/d since onset)	86	140	80	310		-			60	65	30	120		-							1400	**<0.001**		
Previous withdrawal treatments (*n*)	89	6	2	12		-			58	5	2	11		-							2547	0.892		
Alcohol concentration at admission (‰)	108	1.7	0.5	2.4		-			85	1.2	0.1	1.8		-							3696	**0.020**		
AUDIT score		-			125	4	3	6		-			96	3	2	4							4295	**<0.001**
Active smokers (%)	104	78			133	22			78	77			107	19			74	**<0.001**	62	**<0.001**	<1	0.878	<1	0.552
Active and ex-smokers (%)	101	92			133	59			75	85			105	48			31	**<0.001**	27	**<0.001**	2	0.154	3	0.070
CDT (nephelometry, %)	113	2.8	1.9	4.0	132	1.5	1.3	1.7	87	1.9	1.6	2.5	107	1.5	1.3	1.6	1636	**<0.001**	1415	**<0.001**	3003	**<0.001**	6692	0.486
*12-month alcohol-related readmissions*																								
Readmission rate (%)	113	58				-			87	41				-							5	**0.024**		
Latency (days)	113	285	57	≥365		-			87	≥365	97	≥365		-							4142	**0.042**		
Total number	113	1	0	3		-			87	0	0	2		-							4175	0.051		
*24-month alcohol-related readmissions*																								
Readmission rate (%)	113	67				-			87	53				-							4	**0.039**		
Latency (days)	113	285	57	≥730		-			87	625	90	≥730		-							4136	**0.047**		
Total number	113	2	0	4		-			87	1	0	3		-							4014	**0.021**		

The table shows medians (M) or relative frequencies (F), interquartile ranges (IQR), and results of Mann-Whitney *U* tests or χ^2^ tests. AUDIT: Alcohol Use Disorders Identification Test; CDT: carbohydrate-deficient transferrin. *P* < 0.05 in bold.

**Table 2:** Differences in body measurements between alcohol-dependent in-patients (ADP) and healthy control subjects (HCS) and between males and females (Mann-Whitney *U* tests).

	Males								Females								ADP versus HCS				Males versus Females			
	ADP				HCS				ADP				HCS				Males		Females		ADP		HCS	
	*N*	M	IQR		*N*	M	IQR		*N*	M	IQR		*N*	M	IQR		*U*	*P*	*U*	*P*	*U*	*P*	*U*	*P*
**Absolute measure**																								
Body height	107	176.8	172.0	181.0	133	179.1	173.8	184.1	77	164.5	162.0	169.0	107	165.6	161.5	169.1	5668	0.007	4064	0.876	943	<0.001 *	864	<0.001 *
Body weight	106	79.3	68.7	86.6	133	87.4	81.0	97.2	77	66.7	59.8	79.8	106	68.0	59.3	79.3	4115	<0.001 *	4078	0.993	2484	<0.001 *	2260	<0.001 *
Head circumference	107	57.5	56.2	58.9	133	58.4	57.2	59.4	79	55.0	54.0	56.5	107	56.0	54.5	57.3	5436	0.002	3198	0.005	1348	<0.001 *	2626	<0.001 *
Bitragion head arc	107	36.7	35.6	37.5	133	37.4	36.5	38.7	79	35.5	34.5	36.3	107	36.0	35.0	37.4	5149	<0.001	3318	0.012	2444	<0.001 *	4149	<0.001 *
Sagittal head arc	107	36.0	35.0	37.7	133	35.6	34.5	37.0	79	35.0	34.0	36.1	107	34.5	33.0	36.0	6319	0.136	3900	0.367	2814	<0.001	5030	<0.001
Mandibular arc	106	24.0	23.0	25.2	133	24.0	22.5	26.0	77	22.2	21.3	23.0	106	22.4	21.0	23.6	6905	0.785	3907	0.621	1501	<0.001 *	3694	<0.001 *
Lip-chin distance	106	5.5	4.9	6.0	132	5.6	5.4	6.0	79	5.0	4.4	5.5	107	5.0	4.7	5.5	5303	0.001	3446	0.031	2731	<0.001 *	3758	<0.001 *
Wrist circumference	102	17.7	16.8	18.4	128	17.9	17.2	18.6	75	16.0	15.5	16.8	103	15.8	14.9	16.7	5761	0.126	3382	0.156	1037	<0.001 *	1825	<0.001 *
Waist circumference	106	93.0	87.8	103.5	133	96.0	89.5	104.8	77	87.0	78.0	93.5	107	84.0	72.7	92.7	6198	0.109	3565	0.119	2545	<0.001 *	3498	<0.001 *
Hip circumference	106	94.2	88.8	100.0	133	102.0	98.0	107.0	77	96.0	90.8	106.0	107	101.2	95.0	106.8	3076	<0.001 *	3130	0.005	3424	0.063	6342	0.148
Thigh circumference	106	55.2	50.8	58.5	133	60.5	57.8	64.6	79	56.5	53.7	62.0	106	60.5	57.0	64.0	3043	<0.001 *	3000	0.001	3294	0.013	6627	0.426
Calf circumference	106	36.8	35.4	38.9	132	39.5	37.7	41.5	79	36.0	33.8	39.2	107	37.3	35.0	39.5	3785	<0.001 *	3647	0.110	3657	0.141	3999	<0.001 *
Ankle circumference	106	26.2	25.3	27.2	131	26.6	25.9	27.9	79	24.2	23.1	25.8	107	24.6	23.6	25.4	5720	0.020	4107	0.741	1922	<0.001 *	2313	<0.001 *
Foot length	99	26.1	25.1	27.0	105	26.9	26.0	27.6	77	24.0	23.1	24.7	100	24.1	23.5	25.0	3625	<0.001	3413	0.195	805	<0.001 *	733	<0.001 *
Foot breadth	100	9.7	9.4	10.1	107	10.1	9.8	10.4	78	9.1	8.8	9.5	101	9.2	8.9	9.4	3375	<0.001 *	3930	0.978	1559	<0.001 *	725	<0.001 *
**Measure divided by body height**																								
Head circumference	107	0.33	0.32	0.33	133	0.32	0.32	0.33	77	0.33	0.32	0.34	107	0.34	0.33	0.35	6846	0.614	3142	0.006	3036	0.002	3505	<0.001 *
Bitragion head arc	107	0.21	0.20	0.21	133	0.21	0.20	0.22	77	0.22	0.21	0.22	107	0.22	0.21	0.23	6394	0.177	3251	0.015	2492	<0.001 *	3940	<0.001 *
Sagittal head arc	107	0.21	0.20	0.21	133	0.20	0.19	0.21	77	0.21	0.20	0.22	107	0.21	0.20	0.22	5313	0.001	3933	0.601	2912	0.001	3829	<0.001 *
Mandibular arc	106	0.14	0.13	0.14	133	0.14	0.13	0.14	75	0.13	0.13	0.14	106	0.14	0.13	0.14	6665	0.470	3731	0.482	3551	0.222	6906	0.788
Lip-chin distance	106	0.03	0.03	0.03	132	0.03	0.03	0.03	77	0.03	0.03	0.03	107	0.03	0.03	0.03	5843	0.029	3299	0.021	3634	0.206	6382	0.200
Wrist circumference	102	0.10	0.10	0.10	128	0.10	0.10	0.10	73	0.10	0.09	0.10	103	0.10	0.09	0.10	6366	0.746	3343	0.211	2761	0.004	4722	<0.001
Waist circumference	106	0.53	0.50	0.58	133	0.53	0.50	0.59	75	0.52	0.47	0.57	107	0.51	0.44	0.57	6702	0.513	3544	0.180	3654	0.355	5569	0.004
Hip circumference	106	0.53	0.50	0.56	133	0.57	0.54	0.60	75	0.59	0.54	0.64	107	0.61	0.57	0.66	3669	<0.001 *	3095	0.009	1974	<0.001 *	4140	<0.001 *
Thigh circumference	106	0.31	0.29	0.33	133	0.34	0.32	0.36	77	0.34	0.33	0.37	106	0.36	0.34	0.39	3603	<0.001 *	2976	0.002	1868	<0.001 *	4234	<0.001 *
Calf circumference	106	0.21	0.20	0.22	132	0.22	0.21	0.23	77	0.22	0.21	0.24	107	0.23	0.21	0.24	4762	<0.001 *	3492	0.078	3078	0.005	6078	0.064
Ankle circumference	106	0.15	0.14	0.15	131	0.15	0.14	0.15	77	0.15	0.14	0.15	107	0.15	0.14	0.16	6535	0.437	3943	0.619	4036	0.898	6769	0.650
Foot length	99	0.15	0.14	0.15	105	0.15	0.15	0.15	74	0.14	0.14	0.15	100	0.15	0.14	0.15	4526	0.111	2843	0.009	2525	<0.001	4158	0.010
Foot breadth	100	0.06	0.05	0.06	107	0.06	0.05	0.06	75	0.06	0.05	0.06	101	0.06	0.05	0.06	4296	0.014	3759	0.932	3629	0.715	4594	0.062
**Measure residualized on body height**																								
Head circumference	107	0.08	−0.46	0.66	133	0.26	−0.19	0.73	77	−0.46	−1.00	0.24	107	−0.15	−0.53	0.60	6241	0.102	2976	0.001	2674	<0.001 *	5615	0.005
Bitragion head arc	107	−0.09	−0.75	0.30	133	0.25	−0.44	0.91	77	−0.28	−0.99	0.27	107	−0.01	−0.68	0.78	5509	0.003	3195	0.009	3650	0.187	6453	0.215
Sagittal head arc	107	0.15	−0.49	0.78	133	−0.09	−0.80	0.45	77	0.06	−0.62	0.65	107	−0.05	−0.76	0.57	5769	0.012	3783	0.345	3792	0.358	6873	0.649
Mandibular arc	106	0.10	−0.24	0.53	133	0.13	−0.57	0.75	75	−0.22	−0.54	0.15	106	−0.17	−0.73	0.43	6838	0.691	3756	0.527	2843	0.001	5923	0.034
Lip-chin distance	106	0.12	−0.83	0.77	132	0.29	−0.24	0.91	77	−0.37	−1.06	0.28	107	−0.12	−0.80	0.57	5668	0.012	3303	0.022	3288	0.025	5390	0.002
Wrist circumference	102	0.28	−0.11	0.61	128	0.18	−0.10	0.58	73	−0.02	−0.18	0.22	103	−0.13	−0.51	0.32	6409	0.812	3334	0.201	2623	0.001	4477	<0.001 *
Waist circumference	106	−0.01	−0.43	0.63	133	0.10	−0.36	0.84	75	−0.18	−0.88	0.35	107	−0.42	−1.21	0.37	6531	0.329	3517	0.157	3317	0.058	4872	<0.001 *
Hip circumference	106	−0.57	−0.99	−0.02	133	0.19	−0.20	0.63	75	−0.30	−0.90	0.55	107	0.17	−0.42	0.73	3080	<0.001 *	2999	0.004	3238	0.034	6762	0.508
Thigh circumference	106	−0.61	−1.29	−0.11	133	0.20	−0.25	0.79	77	−0.37	−0.82	0.35	106	0.25	−0.26	0.79	3061	<0.001 *	2897	0.001	3065	0.004	6982	0.900
Calf circumference	106	−0.34	−0.83	0.22	132	0.22	−0.23	0.74	77	−0.29	−0.88	0.43	107	0.06	−0.54	0.58	4173	<0.001 *	3527	0.096	3910	0.628	5678	0.009
Ankle circumference	106	0.00	−0.35	0.48	131	0.15	−0.25	0.41	77	−0.22	−0.58	0.15	107	−0.19	−0.45	0.25	6167	0.139	3957	0.647	3229	0.016	5040	<0.001
Foot length	99	−0.02	−0.55	0.72	105	0.23	−0.35	1.08	74	−0.38	−0.97	0.18	100	0.04	−0.65	0.50	4547	0.123	2866	0.011	2628	0.001	4270	0.021
Foot breadth	100	−0.07	−0.69	0.68	107	0.32	−0.15	1.02	75	−0.19	−0.81	0.51	101	−0.14	−0.77	0.36	4056	0.003	3755	0.923	3345	0.222	3389	<0.001 *

Medians (M), interquartile ranges (IQR), absolute body weight in kg, absolute length dimensions in cm, *P* < 0.05 after adjustment using the false discovery rate is colored and indicates the following direction: ADP < HCS, ADP > HCS, males > females, males < females. * *P* < 0.05 after Bonferroni correction.

**Table 3:** Differences in body measurements between alcohol-dependent in-patients (ADP) without and those with prospective 12-month alcohol-related readmission.

	12-Month Readmission in Male ADP										12-Month Readmission in Female ADP									
	No				Yes						No				Yes					
	*N*	M	IQR		*N*	M	IQR		*U*	*P*	*N*	M	IQR		*N*	M	IQR		*U*	*P*
**Absolute measure**																				
Body height	46	177.7	173.0	181.0	61	176.2	171.0	181.4	1200	0.201	45	164.3	160.5	169.0	32	164.8	163.0	169.2	628	0.339
Body weight	45	74.2	68.1	83.3	61	80.2	71.8	90.4	1098	0.079	47	68.2	57.9	76.7	30	65.7	60.5	81.3	650	0.562
Head circumference	46	57.3	56.2	58.0	61	57.5	56.4	59.0	1209	0.221	47	54.5	54.0	56.0	32	55.5	53.7	57.0	593	0.111
Bitragion head arc	46	37.0	36.0	37.7	61	36.3	35.4	37.5	1140	0.097	47	35.5	34.5	36.2	32	35.5	35.0	36.5	640	0.260
Sagittal head arc	46	36.0	35.0	37.4	61	36.0	34.7	37.7	1381	0.890	47	34.8	33.0	36.0	32	35.2	34.1	36.3	608	0.149
Mandibular arc	46	24.2	23.4	25.5	60	23.6	22.8	25.0	1120	0.096	47	22.0	21.0	23.0	30	22.3	22.0	23.1	582	0.194
Lip-chin distance	46	5.5	4.9	6.0	60	5.5	5.0	5.8	1306	0.635	47	5.0	4.2	5.5	32	5.0	4.6	5.5	710	0.668
Wrist circumference	44	17.7	16.5	18.2	58	17.7	16.9	18.5	1131	0.327	46	15.9	15.5	16.8	29	16.1	15.5	16.5	590	0.401
Waist circumference	46	92.0	86.4	97.7	60	95.6	88.3	105.5	1115	0.091	45	87.0	81.0	93.5	32	86.8	77.0	101.0	713	0.938
Hip circumference	46	92.6	88.0	99.4	60	95.3	90.0	100.8	1201	0.252	45	96.5	91.7	103.0	32	95.2	89.8	108.1	717	0.971
Thigh circumference	46	52.9	49.8	56.5	60	56.6	53.5	60.2	911	0.003	47	56.0	53.0	62.5	32	56.9	54.0	61.0	709	0.668
Calf circumference	46	36.2	34.5	37.9	60	37.4	35.8	39.7	998	0.015	47	35.8	33.3	39.2	32	36.8	34.2	39.4	686	0.506
Ankle circumference	46	26.0	25.0	27.0	60	26.5	25.5	27.5	1109	0.084	47	24.1	23.0	26.0	32	24.5	23.8	25.3	672	0.423
Foot length	41	26.0	25.0	27.2	58	26.1	25.3	26.8	1183	0.966	45	23.7	22.7	24.7	32	24.1	23.5	24.8	604	0.229
Foot breadth	42	9.7	9.5	10.0	58	9.7	9.4	10.2	1180	0.788	46	9.1	8.8	9.5	32	9.1	8.9	9.5	731	0.959
**Measure divided by body height**																				
Head circumference	46	0.32	0.31	0.33	61	0.33	0.32	0.34	1071	0.036	45	0.33	0.32	0.34	32	0.33	0.32	0.35	705	0.877
Bitragion head arc	46	0.21	0.20	0.21	61	0.21	0.20	0.22	1304	0.531	45	0.22	0.21	0.22	32	0.22	0.21	0.22	705	0.873
Sagittal head arc	46	0.20	0.20	0.21	61	0.21	0.20	0.21	1285	0.456	45	0.21	0.20	0.22	32	0.21	0.20	0.22	694	0.784
Mandibular arc	46	0.14	0.13	0.15	60	0.14	0.13	0.14	1255	0.426	45	0.13	0.13	0.14	30	0.13	0.13	0.14	628	0.611
Lip-chin distance	46	0.03	0.03	0.03	60	0.03	0.03	0.03	1357	0.883	45	0.03	0.03	0.03	32	0.03	0.03	0.03	689	0.749
Wrist circumference	44	0.10	0.09	0.10	58	0.10	0.10	0.10	1034	0.102	44	0.10	0.09	0.10	29	0.10	0.09	0.10	620	0.839
Waist circumference	46	0.51	0.49	0.56	60	0.54	0.51	0.59	1026	0.024	43	0.54	0.49	0.57	32	0.51	0.46	0.59	663	0.785
Hip circumference	46	0.52	0.49	0.56	60	0.54	0.52	0.57	1076	0.052	43	0.60	0.54	0.64	32	0.57	0.54	0.64	660	0.764
Thigh circumference	46	0.30	0.28	0.32	60	0.32	0.30	0.34	811	<0.001	45	0.34	0.33	0.37	32	0.34	0.33	0.37	690	0.753
Calf circumference	46	0.21	0.19	0.22	60	0.21	0.20	0.23	939	0.005	45	0.22	0.20	0.24	32	0.22	0.21	0.24	677	0.653
Ankle circumference	46	0.15	0.14	0.15	60	0.15	0.14	0.16	1017	0.021	45	0.15	0.14	0.15	32	0.15	0.14	0.15	714	0.946
Foot length	41	0.15	0.14	0.15	58	0.15	0.14	0.15	1050	0.323	42	0.14	0.14	0.15	32	0.15	0.14	0.15	612	0.513
Foot breadth	42	0.05	0.05	0.06	58	0.06	0.05	0.06	1092	0.379	43	0.06	0.05	0.06	32	0.06	0.05	0.06	603	0.363
**Measure residualized on body height**																				
Head circumference	46	−0.14	−0.50	0.40	61	0.23	−0.38	0.76	1104	0.059	45	−0.58	−1.02	0.07	32	−0.34	−0.90	0.39	609	0.251
Bitragion head arc	46	0.01	−0.45	0.31	61	−0.26	−0.99	0.29	1173	0.147	45	−0.49	−0.99	0.32	32	−0.23	−0.95	0.27	677	0.653
Sagittal head arc	46	0.15	−0.44	0.78	61	0.16	−0.54	0.77	1351	0.741	45	−0.02	−0.62	0.62	32	0.08	−0.58	0.69	637	0.391
Mandibular arc	46	0.12	−0.21	0.74	60	0.05	−0.35	0.38	1209	0.276	45	−0.33	−0.69	0.17	30	−0.18	−0.46	0.15	610	0.482
Lip-chin distance	46	0.07	−0.86	1.02	60	0.13	−0.67	0.52	1345	0.823	45	−0.36	−1.23	0.28	32	−0.39	−0.87	0.35	681	0.687
Wrist circumference	44	0.05	−0.18	0.52	58	0.33	−0.03	0.66	1040	0.111	44	−0.04	−0.16	0.24	29	0.01	−0.40	0.22	628	0.910
Waist circumference	46	−0.19	−0.49	0.34	60	0.10	−0.31	0.89	1045	0.032	43	−0.05	−0.60	0.34	32	−0.30	−0.97	0.69	664	0.793
Hip circumference	46	−0.68	−1.11	−0.07	60	−0.42	−0.92	0.09	1189	0.222	43	−0.28	−0.90	0.37	32	−0.38	−0.87	0.77	682	0.949
Thigh circumference	46	−0.98	−1.43	−0.42	60	−0.42	−0.82	0.14	891	0.002	45	−0.42	−0.84	0.51	32	−0.30	−0.73	0.34	677	0.653
Calf circumference	46	−0.61	−1.21	0.01	60	−0.22	−0.60	0.40	964	0.008	45	−0.42	−0.92	0.43	32	−0.09	−0.87	0.44	663	0.552
Ankle circumference	46	−0.10	−0.39	0.28	60	0.15	−0.26	0.68	1041	0.031	45	−0.22	−0.64	0.20	32	−0.21	−0.53	0.13	704	0.865
Foot length	41	−0.03	−0.79	0.51	58	0.09	−0.48	0.86	1054	0.338	42	−0.44	−0.93	0.09	32	−0.07	−1.19	0.21	622	0.585
Foot breadth	42	−0.40	−0.69	0.72	58	0.10	−0.73	0.66	1108	0.442	43	−0.19	−0.70	0.54	32	−0.31	−0.94	0.27	623	0.486

Medians (M), interquartile ranges (IQR), absolute body weight in kg, absolute length dimensions in cm, *P* < 0.05 after adjustment using the false discovery rate is colored and indicates the following direction: ADP without 12-month readmission < ADP with 12-month readmission (no *P* < 0.05 after Bonferroni correction).

**Table 4:** Differences in body measurements between alcohol-dependent in-patients (ADP) without and those with prospective 24-month alcohol-related readmission.

	24-Month Readmission in Male ADP										24-Month Readmission in Female ADP									
	No				Yes						No				Yes					
	*N*	M	IQR		*N*	M	IQR		*U*	*P*	*N*	M	IQR		*N*	M	IQR		*U*	*P*
**Absolute measure**																				
Body height	36	178.1	174.4	181.2	71	176.2	171.0	181.0	973	0.044	35	164.1	160.0	169.2	42	165.0	163.0	169.0	600	0.167
Body weight	35	74.2	67.9	81.9	71	80.0	69.1	89.9	1034	0.161	37	67.2	57.9	76.7	40	66.6	61.2	80.5	675	0.507
Head circumference	36	57.3	56.1	58.0	71	57.5	56.3	59.0	1135	0.343	37	54.8	54.0	56.0	42	55.2	53.4	56.8	707	0.490
Bitragion head arc	36	37.0	36.0	37.8	71	36.5	35.5	37.5	1077	0.184	37	35.5	34.3	36.0	42	35.5	35.0	36.5	666	0.274
Sagittal head arc	36	35.9	34.9	37.6	71	36.0	35.0	37.7	1213	0.665	37	35.0	33.1	36.0	42	35.1	34.0	36.3	691	0.397
Mandibular arc	36	24.4	23.5	25.5	70	24.0	22.6	25.0	977	0.058	37	22.0	21.0	23.0	40	22.3	21.4	23.1	648	0.345
Lip-chin distance	36	5.5	4.9	6.1	70	5.5	4.9	5.8	1099	0.280	37	5.0	4.2	5.5	42	5.0	4.4	5.5	765	0.901
Wrist circumference	34	17.6	16.5	18.2	68	17.7	16.9	18.5	1046	0.433	36	15.8	15.2	16.8	39	16.0	15.5	16.5	613	0.342
Waist circumference	36	92.0	87.3	97.4	70	95.2	88.0	105.0	1058	0.178	35	87.0	76.8	93.5	42	86.8	78.0	94.0	698	0.701
Hip circumference	36	92.6	88.3	99.4	70	95.3	89.2	100.5	1112	0.322	35	96.5	89.0	106.0	42	96.0	90.8	106.0	727	0.931
Thigh circumference	36	52.9	50.0	56.8	70	56.1	52.0	60.0	934	0.029	37	56.0	53.1	62.0	42	56.6	54.0	61.0	738	0.702
Calf circumference	36	36.2	34.3	38.0	70	37.0	35.8	39.3	999	0.081	37	36.0	33.8	39.2	42	36.2	34.0	39.0	762	0.883
Ankle circumference	36	26.0	25.3	27.0	70	26.3	25.4	27.4	1131	0.387	37	24.0	23.0	26.0	42	24.5	23.8	25.5	699	0.440
Foot length	33	26.2	25.2	27.2	66	26.0	25.1	26.8	1013	0.570	35	23.6	22.5	24.7	42	24.1	23.1	25.0	581	0.114
Foot breadth	34	9.7	9.4	10.0	66	9.8	9.4	10.2	999	0.371	36	9.1	8.7	9.5	42	9.1	8.9	9.5	732	0.806
**Measure divided by body height**																				
Head circumference	36	0.32	0.31	0.33	71	0.33	0.32	0.34	906	0.014	35	0.34	0.32	0.34	42	0.33	0.32	0.34	667	0.483
Bitragion head arc	36	0.21	0.20	0.21	71	0.21	0.20	0.22	1231	0.757	35	0.22	0.21	0.22	42	0.22	0.21	0.22	706	0.763
Sagittal head arc	36	0.20	0.20	0.21	71	0.21	0.20	0.21	1055	0.141	35	0.21	0.20	0.23	42	0.21	0.20	0.22	686	0.613
Mandibular arc	36	0.14	0.13	0.15	70	0.14	0.13	0.14	1133	0.397	35	0.13	0.13	0.14	40	0.13	0.13	0.14	675	0.787
Lip-chin distance	36	0.03	0.03	0.03	70	0.03	0.03	0.03	1194	0.660	35	0.03	0.03	0.03	42	0.03	0.03	0.03	720	0.874
Wrist circumference	34	0.10	0.09	0.10	68	0.10	0.10	0.10	894	0.063	34	0.10	0.09	0.10	39	0.10	0.09	0.10	638	0.782
Waist circumference	36	0.51	0.48	0.55	70	0.54	0.50	0.59	948	0.037	33	0.54	0.47	0.57	42	0.51	0.47	0.58	669	0.794
Hip circumference	36	0.52	0.49	0.56	70	0.54	0.52	0.57	978	0.059	33	0.60	0.54	0.64	42	0.58	0.54	0.63	653	0.669
Thigh circumference	36	0.30	0.28	0.32	70	0.32	0.30	0.34	787	0.002	35	0.34	0.33	0.37	42	0.34	0.33	0.37	686	0.613
Calf circumference	36	0.20	0.19	0.22	70	0.21	0.20	0.23	893	0.014	35	0.22	0.20	0.24	42	0.22	0.21	0.23	718	0.858
Ankle circumference	36	0.15	0.14	0.15	70	0.15	0.14	0.16	978	0.059	35	0.15	0.14	0.15	42	0.15	0.14	0.15	727	0.931
Foot length	33	0.15	0.14	0.15	66	0.15	0.14	0.15	929	0.235	32	0.14	0.14	0.15	42	0.15	0.14	0.15	550	0.183
Foot breadth	34	0.05	0.05	0.06	66	0.06	0.05	0.06	866	0.062	33	0.06	0.05	0.06	42	0.06	0.05	0.06	616	0.411
**Measure residualized on body height**																				
Head circumference	36	−0.14	−0.48	0.37	71	0.20	−0.41	0.79	1005	0.071	35	−0.58	−1.02	0.24	42	−0.41	−1.00	0.25	712	0.810
Bitragion head arc	36	0.01	−0.60	0.31	71	−0.26	−0.93	0.30	1140	0.363	35	−0.50	−1.03	0.34	42	−0.23	−0.90	0.18	698	0.701
Sagittal head arc	36	−0.16	−0.54	0.80	71	0.17	−0.34	0.77	1150	0.399	35	0.02	−0.70	0.65	42	0.06	−0.58	0.68	711	0.806
Mandibular arc	36	0.17	−0.21	0.73	70	0.05	−0.46	0.40	1079	0.227	35	−0.33	−0.64	0.17	40	−0.18	−0.49	0.15	653	0.614
Lip-chin distance	36	0.11	−0.85	1.03	70	0.12	−0.76	0.54	1160	0.505	35	−0.35	−1.18	0.28	42	−0.45	−1.05	0.39	723	0.898
Wrist circumference	34	−0.02	−0.20	0.51	68	0.31	−0.02	0.65	907	0.077	34	−0.01	−0.18	0.28	39	−0.04	−0.26	0.22	645	0.842
Waist circumference	36	−0.18	−0.54	0.32	70	0.06	−0.37	0.86	970	0.053	33	−0.04	−0.88	0.34	42	−0.28	−0.88	0.58	676	0.852
Hip circumference	36	−0.73	−1.09	−0.08	70	−0.43	−0.94	0.08	1104	0.296	33	−0.35	−0.96	0.41	42	−0.30	−0.76	0.64	689	0.966
Thigh circumference	36	−0.98	−1.43	−0.41	70	−0.48	−1.06	0.08	914	0.021	35	−0.42	−0.84	0.51	42	−0.32	−0.74	0.35	697	0.694
Calf circumference	36	−0.62	−1.21	0.04	70	−0.28	−0.62	0.35	946	0.036	35	−0.35	−1.05	0.61	42	−0.19	−0.88	0.38	735	0.996
Ankle circumference	36	−0.05	−0.37	0.31	70	0.07	−0.27	0.58	1034	0.131	35	−0.21	−0.70	0.29	42	−0.23	−0.49	0.13	720	0.874
Foot length	33	−0.09	−0.81	0.40	66	0.12	−0.48	0.92	928	0.232	32	−0.45	−0.95	−0.01	42	0.04	−1.13	0.23	561	0.226
Foot breadth	34	−0.48	−0.69	0.49	66	0.12	−0.63	0.73	888	0.089	33	−0.21	−0.70	0.62	42	−0.16	−0.87	0.28	651	0.654

Medians (M), interquartile ranges (IQR), absolute body weight in kg, absolute length dimensions in cm, *P* < 0.05 after adjustment using the false discovery rate is colored and indicates the following direction: ADP without 24-month readmission < ADP with 24-month readmission (no *P* < 0.05 after Bonferroni correction).

**Table 5:** Spearman correlations of absolute body measurements with days to first and number of alcohol-related readmissions in alcohol-dependent in-patients (ADP).

		Male ADP 12-Month Readmission				Male ADP 24-Month Readmission					Female ADP 12-Month Readmission				Female ADP 24-Month Readmission			
		Latency (Days)		Number		Latency (Days)		Number			Latency (Days)		Number		Latency (Days)		Number	
	*N*	ρ	*P*	ρ	*P*	ρ	*P*	ρ	*P*	*N*	ρ	*P*	ρ	*P*	ρ	*P*	ρ	*P*
**Absolute measure**																		
Body height	107	0.091	0.349	−0.082	0.400	0.122	0.210	−0.131	0.179	77	−0.041	0.722	0.123	0.286	−0.098	0.399	0.152	0.187
Body weight	106	−0.150	0.126	0.175	0.072	−0.140	0.152	0.188	0.054	77	−0.028	0.807	0.102	0.379	−0.062	0.592	0.104	0.368
Head circumference	107	−0.131	0.178	0.140	0.151	−0.126	0.196	0.160	0.099	79	−0.132	0.245	0.167	0.141	−0.090	0.433	0.149	0.189
Bitragion head arc	107	0.195	0.044	−0.179	0.065	0.190	0.050	−0.147	0.131	79	−0.058	0.614	0.073	0.525	−0.037	0.743	0.099	0.385
Sagittal head arc	107	−0.006	0.952	0.120	0.217	−0.019	0.848	0.162	0.095	79	−0.178	0.116	0.206	0.069	−0.169	0.137	0.192	0.090
Mandibular arc	106	0.122	0.212	−0.141	0.149	0.141	0.151	−0.142	0.147	77	−0.078	0.503	0.115	0.319	−0.058	0.618	0.102	0.376
Lip-chin distance	106	0.040	0.686	−0.094	0.339	0.060	0.542	−0.133	0.174	79	−0.040	0.724	0.002	0.986	0.008	0.945	−0.020	0.865
Wrist circumference	102	−0.121	0.225	0.110	0.270	−0.116	0.244	0.120	0.230	75	−0.049	0.679	0.103	0.378	−0.096	0.411	0.111	0.342
Waist circumference	106	−0.106	0.279	0.142	0.147	−0.096	0.326	0.146	0.135	77	0.043	0.708	0.011	0.928	0.010	0.930	0.044	0.706
Hip circumference	106	−0.095	0.334	0.127	0.196	−0.091	0.351	0.151	0.123	77	0.050	0.666	−0.012	0.920	0.035	0.761	−0.029	0.801
Thigh circumference	106	−0.260	0.007	0.274	0.004	−0.240	0.013	0.257	0.008	79	−0.055	0.628	0.118	0.301	−0.080	0.484	0.095	0.405
Calf circumference	106	−0.270	0.005	0.199	0.041	−0.249	0.010	0.181	0.063	79	−0.050	0.660	0.124	0.276	−0.050	0.664	0.060	0.598
Ankle circumference	106	−0.123	0.210	0.140	0.153	−0.097	0.322	0.087	0.376	79	−0.062	0.588	0.118	0.302	−0.090	0.431	0.094	0.408
Foot length	99	−0.030	0.765	0.006	0.953	−0.009	0.929	−0.026	0.797	77	−0.088	0.449	0.185	0.108	−0.150	0.193	0.215	0.061
Foot breadth	100	−0.110	0.276	0.039	0.700	−0.131	0.195	0.092	0.363	78	0.062	0.588	0.002	0.987	0.015	0.899	−0.004	0.974
**Measure divided by body height**																		
Head circumference	107	−0.184	0.058	0.200	0.038	−0.203	0.036	0.260	0.007	77	−0.044	0.702	−0.016	0.891	0.024	0.837	−0.048	0.676
Bitragion head arc	107	0.108	0.267	−0.101	0.300	0.079	0.416	−0.040	0.686	77	0.013	0.911	−0.060	0.605	0.058	0.614	−0.051	0.660
Sagittal head arc	107	−0.040	0.684	0.145	0.136	−0.069	0.478	0.224	0.020	77	−0.100	0.386	0.060	0.605	−0.054	0.642	0.026	0.824
Mandibular arc	106	0.041	0.674	−0.079	0.423	0.050	0.612	−0.064	0.517	75	−0.031	0.792	0.025	0.833	0.000	0.997	0.010	0.931
Lip-chin distance	106	0.022	0.826	−0.063	0.520	0.030	0.764	−0.083	0.397	77	−0.047	0.687	0.000	0.999	0.002	0.985	−0.019	0.869
Wrist circumference	102	−0.175	0.079	0.159	0.109	−0.187	0.060	0.187	0.060	73	0.034	0.776	0.010	0.933	0.010	0.934	−0.004	0.973
Waist circumference	106	−0.151	0.123	0.183	0.060	−0.149	0.128	0.192	0.049	75	0.065	0.577	−0.033	0.777	0.064	0.584	−0.022	0.852
Hip circumference	106	−0.161	0.100	0.180	0.064	−0.164	0.093	0.211	0.030	75	0.054	0.644	−0.037	0.750	0.052	0.659	−0.062	0.598
Thigh circumference	106	−0.308	0.001	0.315	0.001	−0.303	0.002	0.320	0.001	77	0.009	0.935	0.040	0.729	−0.001	0.994	0.003	0.979
Calf circumference	106	−0.284	0.003	0.212	0.029	−0.277	0.004	0.223	0.022	77	−0.047	0.685	0.098	0.395	−0.034	0.769	0.023	0.845
Ankle circumference	106	−0.181	0.063	0.178	0.068	−0.172	0.079	0.153	0.117	77	−0.017	0.884	0.038	0.740	−0.028	0.809	0.002	0.984
Foot length	99	−0.122	0.230	0.090	0.374	−0.129	0.201	0.103	0.312	74	−0.086	0.464	0.129	0.272	−0.159	0.175	0.177	0.131
Foot breadth	100	−0.142	0.159	0.084	0.407	−0.178	0.076	0.163	0.105	75	0.115	0.325	−0.098	0.402	0.100	0.391	−0.121	0.300
**Measure residualized on body height**																		
Head circumference	107	−0.184	0.058	0.196	0.043	−0.187	0.054	0.232	0.016	77	−0.115	0.320	0.112	0.331	−0.058	0.614	0.089	0.439
Bitragion head arc	107	0.183	0.059	−0.171	0.078	0.170	0.080	−0.120	0.217	77	−0.016	0.891	0.002	0.986	0.019	0.871	0.020	0.864
Sagittal head arc	107	−0.021	0.832	0.129	0.184	−0.042	0.669	0.187	0.054	77	−0.134	0.246	0.136	0.237	−0.114	0.324	0.123	0.287
Mandibular arc	106	0.068	0.487	−0.099	0.311	0.080	0.412	−0.089	0.366	75	−0.038	0.746	0.043	0.716	−0.015	0.900	0.031	0.793
Lip-chin distance	106	0.023	0.812	−0.069	0.485	0.037	0.705	−0.095	0.331	77	−0.051	0.659	0.005	0.967	−0.001	0.995	−0.017	0.885
Wrist circumference	102	−0.171	0.085	0.158	0.113	−0.181	0.068	0.183	0.066	73	0.023	0.846	0.021	0.857	−0.001	0.994	0.006	0.956
Waist circumference	106	−0.144	0.140	0.178	0.068	−0.141	0.149	0.187	0.056	75	0.069	0.556	−0.026	0.824	0.058	0.620	−0.012	0.919
Hip circumference	106	−0.101	0.302	0.132	0.177	−0.097	0.322	0.155	0.114	75	0.036	0.756	−0.001	0.990	0.020	0.866	−0.017	0.883
Thigh circumference	106	−0.269	0.005	0.282	0.003	−0.249	0.010	0.267	0.006	77	−0.058	0.614	0.125	0.280	−0.083	0.471	0.101	0.380
Calf circumference	106	−0.280	0.004	0.209	0.032	−0.265	0.006	0.204	0.036	77	−0.049	0.671	0.117	0.311	−0.043	0.708	0.047	0.686
Ankle circumference	106	−0.165	0.092	0.166	0.089	−0.147	0.132	0.125	0.202	77	−0.018	0.875	0.052	0.652	−0.042	0.717	0.026	0.822
Foot length	99	−0.118	0.244	0.088	0.387	−0.127	0.210	0.102	0.314	74	−0.076	0.521	0.117	0.320	−0.147	0.212	0.166	0.158
Foot breadth	100	−0.139	0.167	0.080	0.431	−0.173	0.085	0.156	0.122	75	0.099	0.397	−0.067	0.571	0.068	0.562	−0.078	0.508

*P* < 0.05 after adjustment using the false discovery rate is colored and indicates the following direction: higher values of body measurements correspond to a shorter latency to the first readmission and to a higher number of readmissions for the 12- and 24-month follow-up periods (no *P* < 0.05 after Bonferroni correction).

## Abbreviations

2D:4D	Second-to-fourth finger length ratio
CDT	Carbohydrate-deficient transferrin
DSM-5	Fifth edition of the Diagnostic and Statistical Manual of Mental Disorders
FASD	Fetal alcohol spectrum disorder
FDR	False discovery rate
ICD-10	Tenth revision of the International Classification of Diseases
IQR	Interquartile range
TTT	Twin testosterone transfer model
WHO	World Health Organization

## Appendix A

**Table A1:** Spearman correlations of body measurements with 2D:4D in male and female alcohol-dependent in-patients (ADP) and healthy control subjects (HCS).

	Males						Females					
	ADP			HCS			ADP			HCS		
	*N*	ρ	*P*	*N*	ρ	*P*	*N*	ρ	*P*	*N*	ρ	*P*
**Absolute measure**												
Body height	103	0.049	0.625	133	−0.068	0.439	76	−0.016	0.894	105	0.090	0.360
Body weight	102	−0.112	0.261	133	−0.059	0.501	76	−0.104	0.369	104	0.006	0.955
Head circumference	103	0.011	0.914	133	−0.109	0.210	78	0.060	0.603	105	−0.038	0.703
Bitragion head arc	103	0.003	0.979	133	0.062	0.480	78	0.008	0.944	105	−0.015	0.878
Sagittal head arc	103	0.007	0.943	133	−0.048	0.587	78	0.029	0.802	105	−0.057	0.564
Mandibular arc	102	−0.066	0.512	133	−0.056	0.526	76	−0.130	0.264	104	0.112	0.259
Lip-chin distance	102	−0.021	0.832	132	−0.034	0.702	78	−0.069	0.549	105	−0.092	0.351
Wrist circumference	98	−0.156	0.126	128	−0.091	0.308	74	0.061	0.609	101	−0.124	0.218
Waist circumference	102	−0.143	0.153	133	−0.131	0.134	76	−0.017	0.886	105	0.026	0.789
Hip circumference	102	−0.187	0.060	133	−0.086	0.325	76	−0.074	0.523	105	−0.024	0.810
Thigh circumference	102	−0.052	0.601	133	−0.086	0.323	78	−0.119	0.297	104	−0.059	0.549
Calf circumference	102	−0.082	0.412	132	−0.046	0.599	78	−0.055	0.635	105	0.065	0.508
Ankle circumference	102	−0.041	0.682	131	**−0.176**	**0.044**	78	0.055	0.630	105	0.051	0.603
Foot length	96	0.061	0.556	105	0.074	0.451	76	0.047	0.689	98	0.039	0.702
Foot breadth	97	−0.021	0.842	107	−0.088	0.368	77	0.030	0.798	99	−0.025	0.806
**Measure divided by body height**												
Head circumference	103	−0.037	0.711	133	−0.002	0.985	76	0.022	0.853	105	−0.169	0.085
Bitragion head arc	103	−0.032	0.748	133	0.105	0.227	76	0.010	0.929	105	−0.090	0.361
Sagittal head arc	103	0.000	0.999	133	−0.002	0.981	76	0.049	0.671	105	−0.151	0.124
Mandibular arc	102	−0.092	0.358	133	−0.040	0.649	74	−0.105	0.372	104	0.016	0.875
Lip-chin distance	102	−0.057	0.570	132	−0.027	0.762	76	−0.056	0.634	105	−0.130	0.188
Wrist circumference	98	**−0.204**	**0.044**	128	−0.018	0.836	72	0.059	0.622	101	**−0.198**	**0.048**
Waist circumference	102	−0.153	0.125	133	−0.084	0.335	74	−0.011	0.926	105	−0.019	0.850
Hip circumference	102	**−0.197**	**0.047**	133	−0.039	0.655	74	−0.023	0.845	105	−0.073	0.459
Thigh circumference	102	−0.060	0.549	133	−0.043	0.626	76	−0.093	0.425	104	−0.122	0.217
Calf circumference	102	−0.079	0.431	132	−0.031	0.726	76	−0.004	0.975	105	−0.003	0.979
Ankle circumference	102	−0.090	0.367	131	−0.153	0.082	76	0.079	0.496	105	−0.060	0.546
Foot length	96	−0.043	0.676	105	0.060	0.546	73	0.067	0.573	98	−0.092	0.368
Foot breadth	97	−0.103	0.313	107	−0.087	0.374	74	0.020	0.864	99	−0.097	0.341
**Measure residualized on body height**												
Head circumference	103	−0.003	0.979	133	−0.077	0.378	76	0.043	0.715	105	−0.107	0.276
Bitragion head arc	103	−0.006	0.952	133	0.082	0.349	76	−0.019	0.872	105	−0.042	0.674
Sagittal head arc	103	0.017	0.868	133	−0.018	0.840	76	0.002	0.988	105	−0.095	0.335
Mandibular arc	102	−0.090	0.367	133	−0.046	0.596	74	−0.138	0.241	104	0.050	0.617
Lip-chin distance	102	−0.059	0.556	132	−0.032	0.717	76	−0.054	0.641	105	−0.120	0.221
Wrist circumference	98	**−0.204**	**0.044**	128	−0.021	0.810	72	0.059	0.624	101	**−0.201**	**0.044**
Waist circumference	102	−0.156	0.118	133	−0.101	0.245	74	−0.018	0.882	105	−0.014	0.887
Hip circumference	102	−0.190	0.055	133	−0.086	0.326	74	−0.062	0.602	105	−0.026	0.789
Thigh circumference	102	−0.055	0.585	133	−0.081	0.355	76	−0.121	0.296	104	−0.066	0.503
Calf circumference	102	−0.083	0.407	132	−0.056	0.524	76	−0.044	0.703	105	0.040	0.688
Ankle circumference	102	−0.092	0.357	131	**−0.180**	**0.040**	76	0.069	0.556	105	−0.027	0.787
Foot length	96	−0.048	0.641	105	0.051	0.606	73	0.077	0.519	98	−0.095	0.354
Foot breadth	97	−0.091	0.377	107	−0.091	0.351	74	0.000	0.999	99	−0.098	0.333

*P* < 0.05 in bold. Significant negative correlation.

**Table A2:** Spearman correlations of body measurements with the age of onset of regular alcohol drinking (i.e., daily over at least 7 days) and the age of first in-patient treatment due to alcohol problems in male and female alcohol-dependent in-patients.

	Males: The Age of						Females: The Age of					
	Onset of Regular Alcohol Drinking (i.e., Daily over at Least 7 Days)			First In-Patient Treatment Due to Alcohol Problems			Onset of Regular Alcohol Drinking (i.e., Daily over at Least 7 Days)			First In-Patient Treatment Due to Alcohol Problems		
	*N*	ρ	*P*	*N*	ρ	*P*	*N*	ρ	*P*	*N*	ρ	*P*
**Absolute measure**												
Body height	91	−0.087	0.412	95	−0.043	0.677	61	−0.145	0.265	71	−0.089	0.462
Body weight	91	−0.070	0.510	95	0.133	0.201	61	0.031	0.812	71	0.055	0.650
Head circumference	91	0.039	0.715	95	0.129	0.212	63	−0.015	0.906	73	0.030	0.800
Bitragion head arc	91	0.127	0.231	95	0.110	0.288	63	0.061	0.637	73	0.159	0.180
Sagittal head arc	91	**−0.225**	**0.032**	95	**−0.311**	**0.002**	63	0.056	0.662	73	0.116	0.330
Mandibular arc	90	**0.213**	**0.044**	94	0.202	0.051	61	0.084	0.519	71	0.051	0.674
Lip-chin distance	90	0.011	0.921	94	0.115	0.271	63	0.023	0.861	73	0.044	0.714
Wrist circumference	87	−0.077	0.478	91	0.150	0.157	61	0.132	0.311	70	0.202	0.094
Waist circumference	91	0.016	0.880	95	**0.215**	**0.036**	63	0.124	0.331	71	0.110	0.359
Hip circumference	91	0.061	0.569	95	**0.221**	**0.031**	63	0.024	0.851	71	0.111	0.356
Thigh circumference	91	**−0.222**	**0.035**	95	0.048	0.646	63	−0.028	0.825	73	0.018	0.882
Calf circumference	91	−0.161	0.126	95	0.072	0.485	63	0.046	0.722	73	0.054	0.653
Ankle circumference	91	−0.103	0.333	95	**0.234**	**0.022**	63	−0.093	0.470	73	0.108	0.361
Foot length	84	−0.097	0.382	87	0.090	0.405	62	−0.123	0.339	71	0.025	0.838
Foot breadth	85	−0.049	0.659	88	0.155	0.149	63	0.189	0.137	72	0.180	0.130
**Measure divided by body height**												
Head circumference	91	0.097	0.361	95	0.078	0.451	61	0.170	0.190	71	0.120	0.319
Bitragion head arc	91	0.164	0.121	95	0.136	0.190	61	0.219	0.090	71	0.219	0.067
Sagittal head arc	91	−0.147	0.164	95	**−0.266**	**0.009**	61	0.187	0.149	71	0.193	0.107
Mandibular arc	90	**0.227**	**0.031**	94	**0.206**	**0.047**	59	0.165	0.211	69	0.192	0.114
Lip-chin distance	90	0.043	0.685	94	0.129	0.216	61	0.067	0.610	71	0.099	0.413
Wrist circumference	87	−0.087	0.424	91	0.156	0.140	59	**0.271**	**0.038**	68	**0.339**	**0.005**
Waist circumference	91	0.004	0.973	95	**0.216**	**0.035**	61	0.200	0.122	69	0.208	0.087
Hip circumference	91	0.074	0.486	95	0.195	0.058	61	0.108	0.409	69	0.201	0.098
Thigh circumference	91	**−0.218**	**0.038**	95	0.042	0.684	61	0.108	0.406	71	0.110	0.359
Calf circumference	91	−0.143	0.176	95	0.073	0.482	61	0.133	0.308	71	0.119	0.324
Ankle circumference	91	−0.057	0.590	95	**0.226**	**0.028**	61	0.055	0.671	71	**0.287**	**0.015**
Foot length	84	−0.009	0.939	87	0.174	0.107	59	0.003	0.985	68	**0.300**	**0.013**
Foot breadth	85	−0.024	0.826	88	0.149	0.166	60	**0.321**	**0.012**	69	**0.287**	**0.017**
**Measure residualized on body height**												
Head circumference	91	0.042	0.694	95	0.104	0.317	61	0.085	0.516	71	0.130	0.279
Bitragion head arc	91	0.159	0.133	95	0.127	0.219	61	0.140	0.280	71	**0.234**	**0.050**
Sagittal head arc	91	−0.200	0.058	95	**−0.312**	**0.002**	61	0.097	0.457	71	0.155	0.197
Mandibular arc	90	**0.244**	**0.020**	94	**0.209**	**0.043**	59	0.155	0.242	69	0.158	0.194
Lip-chin distance	90	0.039	0.718	94	0.128	0.219	61	0.058	0.654	71	0.084	0.487
Wrist circumference	87	−0.097	0.369	91	0.154	0.145	59	0.256	0.050	68	**0.332**	**0.006**
Waist circumference	91	−0.012	0.907	95	**0.207**	**0.045**	61	0.198	0.126	69	0.192	0.114
Hip circumference	91	0.069	0.518	95	**0.222**	**0.030**	61	0.055	0.674	69	0.141	0.246
Thigh circumference	91	**−0.216**	**0.040**	95	0.054	0.602	61	0.021	0.872	71	0.070	0.561
Calf circumference	91	−0.144	0.173	95	0.083	0.425	61	0.098	0.453	71	0.100	0.406
Ankle circumference	91	−0.070	0.507	95	**0.229**	**0.026**	61	0.019	0.886	71	**0.260**	**0.028**
Foot length	84	−0.012	0.915	87	0.177	0.101	59	0.014	0.918	68	**0.319**	**0.008**
Foot breadth	85	−0.040	0.713	88	0.149	0.166	60	**0.302**	**0.019**	69	**0.287**	**0.017**

*P* < 0.05 in bold. Significant positive correlation, significant negative correlation.

**Table A3:** Spearman correlations of body measurements with total lifetime drinking and daily ethanol intake in male and female alcohol-dependent in-patients.

	Males						Females					
	Total Lifetime Drinking			Daily Ethanol Intake			Total Lifetime Drinking			Daily Ethanol Intake		
	*N*	ρ	*P*	*N*	ρ	*P*	*N*	ρ	*P*	*N*	ρ	*P*
**Absolute measure**												
Body height	87	0.001	0.993	85	0.142	0.196	57	−0.051	0.705	55	0.060	0.664
Body weight	87	0.073	0.504	85	0.099	0.368	57	0.188	0.162	55	0.075	0.587
Head circumference	87	−0.074	0.493	85	−0.051	0.643	59	−0.046	0.728	57	0.059	0.660
Bitragion head arc	87	0.061	0.575	85	0.059	0.593	59	0.056	0.674	57	0.026	0.847
Sagittal head arc	87	−0.071	0.513	85	0.064	0.561	59	0.198	0.134	57	0.128	0.341
Mandibular arc	86	−0.144	0.186	84	−0.189	0.085	57	−0.115	0.393	55	**−0.272**	**0.045**
Lip-chin distance	86	**−0.228**	**0.035**	84	−0.188	0.086	59	−0.188	0.155	57	−0.080	0.553
Wrist circumference	83	0.018	0.870	81	0.068	0.546	58	0.042	0.756	56	−0.039	0.773
Waist circumference	87	0.097	0.370	85	0.005	0.963	58	0.219	0.099	56	0.134	0.323
Hip circumference	87	0.095	0.383	85	0.072	0.512	58	0.108	0.422	56	0.006	0.963
Thigh circumference	87	−0.035	0.745	85	0.040	0.718	59	0.066	0.618	57	0.103	0.444
Calf circumference	87	−0.019	0.860	85	0.073	0.506	59	0.047	0.721	57	−0.059	0.661
Ankle circumference	87	−0.150	0.166	85	−0.044	0.692	59	0.084	0.526	57	−0.064	0.638
Foot length	79	−0.087	0.444	77	0.108	0.348	57	0.144	0.284	55	0.141	0.306
Foot breadth	80	−0.028	0.808	78	−0.006	0.960	58	0.052	0.701	56	−0.015	0.914
**Measure divided by body height**												
Head circumference	87	−0.058	0.595	85	−0.185	0.091	57	−0.046	0.733	55	−0.038	0.783
Bitragion head arc	87	0.099	0.360	85	−0.054	0.621	57	0.075	0.577	55	−0.009	0.951
Sagittal head arc	87	−0.073	0.501	85	−0.036	0.741	57	0.156	0.248	55	0.070	0.614
Mandibular arc	86	−0.154	0.156	84	**−0.242**	**0.027**	55	−0.129	0.349	53	**−0.301**	**0.029**
Lip-chin distance	86	−0.204	0.059	84	−0.211	0.054	57	−0.201	0.134	55	−0.111	0.419
Wrist circumference	83	0.046	0.681	81	−0.020	0.862	56	0.036	0.794	54	−0.098	0.482
Waist circumference	87	0.114	0.295	85	−0.012	0.913	56	0.182	0.180	54	0.093	0.505
Hip circumference	87	0.129	0.233	85	0.041	0.707	56	0.110	0.419	54	−0.032	0.819
Thigh circumference	87	0.010	0.924	85	0.017	0.874	57	0.000	0.997	55	0.033	0.812
Calf circumference	87	0.000	0.997	85	0.046	0.677	57	0.055	0.686	55	−0.117	0.394
Ankle circumference	87	−0.140	0.196	85	−0.130	0.235	57	0.092	0.495	55	−0.127	0.354
Foot length	79	−0.068	0.549	77	0.021	0.857	54	0.132	0.341	52	0.126	0.375
Foot breadth	80	0.039	0.734	78	−0.093	0.417	55	0.044	0.751	53	−0.117	0.406
**Measure residualized on body height**												
Head circumference	87	−0.077	0.477	85	−0.120	0.275	57	−0.080	0.556	55	0.018	0.898
Bitragion head arc	87	0.069	0.525	85	0.009	0.934	57	0.058	0.666	55	0.003	0.980
Sagittal head arc	87	−0.072	0.509	85	0.007	0.946	57	0.197	0.142	55	0.135	0.327
Mandibular arc	86	−0.151	0.166	84	**−0.226**	**0.039**	55	−0.154	0.261	53	**−0.306**	**0.026**
Lip-chin distance	86	−0.207	0.056	84	−0.189	0.085	57	−0.209	0.119	55	−0.116	0.400
Wrist circumference	83	0.035	0.752	81	−0.013	0.908	56	0.037	0.784	54	−0.094	0.501
Waist circumference	87	0.115	0.290	85	−0.009	0.936	56	0.193	0.154	54	0.119	0.393
Hip circumference	87	0.099	0.361	85	0.067	0.545	56	0.090	0.511	54	0.008	0.954
Thigh circumference	87	−0.025	0.822	85	0.038	0.731	57	0.037	0.784	55	0.091	0.508
Calf circumference	87	−0.003	0.979	85	0.062	0.573	57	0.052	0.700	55	−0.079	0.566
Ankle circumference	87	−0.158	0.144	85	−0.109	0.320	57	0.097	0.473	55	−0.086	0.534
Foot length	79	−0.068	0.554	77	0.020	0.862	54	0.133	0.339	52	0.111	0.434
Foot breadth	80	0.046	0.684	78	−0.067	0.562	55	0.032	0.814	53	−0.107	0.446

*P* < 0.05 in bold. Significant negative correlation.

**Table A4:** Inter-correlations between absolute male body measurements (alcohol-dependent in-patients bottom left, healthy control subjects top right).

*N*	Body height	133	133	133	133	133	132	128	133	133	133	132	131	105	107
ρ		0.264	0.227	0.232	0.295	0.141	0.146	0.189	−0.097	0.090	0.029	0.065	0.335	0.610	0.474
*P*		**0.002**	**0.009**	**0.007**	**0.001**	0.105	0.095	**0.033**	0.269	0.303	0.741	0.461	**<0.001**	**<0.001**	**<0.001**
*N*	106	Body weight	133	133	133	133	132	128	133	133	133	132	131	105	107
ρ	0.368		0.259	0.074	0.026	0.204	0.166	0.626	0.818	0.874	0.698	0.675	0.467	0.213	0.360
*P*	**<0.001**		**0.003**	0.395	0.765	**0.019**	0.057	**<0.001**	**<0.001**	**<0.001**	**<0.001**	**<0.001**	**<0.001**	**0.029**	**<0.001**
*N*	107	106	Head circum-ference	133	133	133	132	128	133	133	133	132	131	105	107
ρ	0.175	0.453		0.500	0.475	0.152	0.271	0.319	0.183	0.229	0.206	0.304	0.408	0.338	0.264
*P*	0.071	**<0.001**		**<0.001**	**<0.001**	0.081	**0.002**	**<0.001**	**0.035**	**0.008**	**0.017**	**<0.001**	**<0.001**	**<0.001**	**0.006**
*N*	107	106	107	Bitragion head arc	133	133	132	128	133	133	133	132	131	105	107
ρ	0.326	0.168	0.390		0.393	−0.013	0.138	0.143	−0.004	0.044	0.038	0.046	0.198	0.351	0.215
*P*	**0.001**	0.085	**<0.001**		**<0.001**	0.880	0.115	0.107	0.961	0.612	0.661	0.600	**0.023**	**<0.001**	**0.026**
*N*	107	106	107	107	Sagittal head arc	133	132	128	133	133	133	132	131	105	107
ρ	0.285	0.031	0.288	0.341		0.040	0.162	0.020	−0.094	0.011	0.081	0.120	0.248	0.219	0.111
*P*	**0.003**	0.749	**0.003**	**<0.001**		0.647	0.063	0.819	0.281	0.901	0.353	0.171	**0.004**	**0.025**	0.255
*N*	106	105	106	106	106	Mandi-bular arc	132	128	133	133	133	132	131	105	107
ρ	0.115	0.411	0.283	0.161	−0.085		0.330	0.303	0.195	0.162	0.089	0.245	0.348	0.213	0.128
*P*	0.239	**<0.001**	**0.003**	0.099	0.389		**<0.001**	**0.001**	**0.024**	0.062	0.306	**0.005**	**<0.001**	**0.029**	0.188
*N*	106	105	106	106	106	105	Lip-chin distance	127	132	132	132	131	130	104	106
ρ	0.001	0.187	0.284	0.094	−0.259	0.397		0.239	0.127	0.140	0.190	0.229	0.201	0.282	0.229
*P*	0.990	0.055	**0.003**	0.339	**0.007**	**<0.001**		**0.007**	0.147	0.110	**0.029**	**0.008**	**0.022**	**0.004**	**0.018**
*N*	102	101	102	102	102	101	101	Wrist circum-ference	128	128	128	127	126	101	103
ρ	0.337	0.692	0.414	0.222	0.001	0.467	0.337		0.503	0.552	0.459	0.483	0.535	0.187	0.361
*P*	**0.001**	**<0.001**	**<0.001**	**0.025**	0.995	**<0.001**	**0.001**		**<0.001**	**<0.001**	**<0.001**	**<0.001**	**<0.001**	0.061	**<0.001**
*N*	106	105	106	106	106	105	105	102	Waist circum-ference	133	133	132	131	105	107
ρ	0.169	0.886	0.395	0.183	−0.081	0.455	0.259	0.569		0.778	0.581	0.531	0.314	0.013	0.135
*P*	0.084	**<0.001**	**<0.001**	0.061	0.407	**<0.001**	**0.008**	**<0.001**		**<0.001**	**<0.001**	**<0.001**	**<0.001**	0.893	0.165
*N*	106	105	106	106	106	105	105	102	106	Hip circum-ference	133	132	131	105	107
ρ	0.263	0.878	0.457	0.228	−0.012	0.435	0.242	0.623	0.839		0.768	0.625	0.377	0.088	0.229
*P*	**0.007**	**<0.001**	**<0.001**	**0.019**	0.905	**<0.001**	**0.013**	**<0.001**	**<0.001**		**<0.001**	**<0.001**	**<0.001**	0.371	**0.018**
*N*	106	105	106	106	106	105	105	102	106	106	Thigh circum-ference	132	131	105	107
ρ	0.160	0.831	0.426	0.012	−0.033	0.428	0.291	0.595	0.725	0.708		0.673	0.319	−0.067	0.157
*P*	0.101	**<0.001**	**<0.001**	0.902	0.739	**<0.001**	**0.003**	**<0.001**	**<0.001**	**<0.001**		**<0.001**	**<0.001**	0.495	0.107
*N*	106	105	106	106	106	105	105	102	106	106	106	Calf circum-ference	131	104	106
ρ	0.126	0.724	0.285	0.077	0.024	0.336	0.155	0.560	0.580	0.569	0.706		0.417	0.054	0.348
*P*	0.199	**<0.001**	**0.003**	0.430	0.807	**<0.001**	0.115	**<0.001**	**<0.001**	**<0.001**	**<0.001**		**<0.001**	0.587	**<0.001**
*N*	106	105	106	106	106	105	105	102	106	106	106	106	Ankle circum-ference	103	105
ρ	0.323	0.626	0.424	0.264	−0.060	0.347	0.381	0.580	0.574	0.578	0.539	0.582		0.497	0.565
*P*	**0.001**	**<0.001**	**<0.001**	**0.006**	0.543	**<0.001**	**<0.001**	**<0.001**	**<0.001**	**<0.001**	**<0.001**	**<0.001**		**<0.001**	**<0.001**
*N*	99	98	99	99	99	98	98	95	98	98	98	98	98	Foot length	105
ρ	0.690	0.497	0.253	0.293	0.239	0.245	0.262	0.480	0.354	0.425	0.354	0.330	0.544		0.527
*P*	**<0.001**	**<0.001**	**0.012**	**0.003**	**0.017**	**0.015**	**0.009**	**<0.001**	**<0.001**	**<0.001**	**<0.001**	**0.001**	**<0.001**		**<0.001**
*N*	100	99	100	100	100	99	99	95	99	99	99	99	99	99	Foot breadth
ρ	0.293	0.563	0.368	0.234	−0.034	0.264	0.253	0.602	0.443	0.482	0.431	0.472	0.636	0.494	
*P*	**0.003**	**<0.001**	**<0.001**	**0.019**	0.739	**0.008**	**0.012**	**<0.001**	**<0.001**	**<0.001**	**<0.001**	**<0.001**	**<0.001**	**<0.001**	

Spearman correlations. *P* < 0.05 in bold.

**Table A5:** Inter-correlations between absolute female body measurements (alcohol-dependent in-patients bottom left, healthy control subjects top right).

*N*	Body height	106	107	107	107	106	107	103	107	107	106	107	107	100	101
ρ		0.168	0.304	0.079	0.288	0.078	0.070	0.196	−0.057	0.051	0.018	0.032	0.336	0.610	0.267
*P*		0.085	**0.001**	0.421	**0.003**	0.429	0.472	**0.047**	0.563	0.603	0.852	0.743	**<0.001**	**<0.001**	**0.007**
*N*	75	Body weight	106	106	106	105	106	102	106	106	105	106	106	99	100
ρ	0.246		0.344	0.020	−0.010	0.500	0.252	0.533	0.842	0.898	0.848	0.821	0.596	0.291	0.510
*P*	**0.034**		**<0.001**	0.841	0.922	**<0.001**	**0.009**	**<0.001**	**<0.001**	**<0.001**	**<0.001**	**<0.001**	**<0.001**	**0.003**	**<0.001**
*N*	77	77	Head circum-ference	107	107	106	107	103	107	107	106	107	107	100	101
ρ	0.234	0.432		0.612	0.433	0.219	0.258	0.220	0.214	0.319	0.312	0.263	0.335	0.330	0.381
*P*	**0.041**	**<0.001**		**<0.001**	**<0.001**	**0.024**	**0.007**	**0.026**	**0.027**	**0.001**	**0.001**	**0.006**	**<0.001**	**0.001**	**<0.001**
*N*	77	77	79	Bitragion head arc	107	106	107	103	107	107	106	107	107	100	101
ρ	0.221	0.190	0.405		0.382	0.142	0.242	−0.003	−0.016	0.015	0.091	0.028	0.139	0.100	0.258
*P*	0.054	0.097	**<0.001**		**<0.001**	0.146	**0.012**	0.978	0.867	0.875	0.351	0.776	0.154	0.323	**0.009**
*N*	77	77	79	79	Sagittal head arc	106	107	103	107	107	106	107	107	100	101
ρ	0.080	0.227	0.352	0.311		−0.052	0.214	−0.094	−0.164	0.010	0.070	−0.044	0.035	0.221	0.173
*P*	0.489	**0.047**	**0.001**	**0.005**		0.595	**0.027**	0.343	0.092	0.920	0.477	0.652	0.721	**0.027**	0.083
*N*	75	75	77	77	77	Mandibu-lar arc	106	103	106	106	105	106	106	99	100
ρ	0.146	0.459	0.278	0.295	0.039		0.304	0.299	0.434	0.508	0.458	0.413	0.433	0.194	0.360
*P*	0.213	**<0.001**	**0.014**	**0.009**	0.738		**0.002**	**0.002**	**<0.001**	**<0.001**	**<0.001**	**<0.001**	**<0.001**	0.055	**<0.001**
*N*	77	77	79	79	79	77	Lip-chin distance	103	107	107	106	107	107	100	101
ρ	−0.103	0.089	0.188	−0.039	−0.443	0.206		0.216	0.276	0.225	0.226	0.167	0.104	0.166	0.099
*P*	0.375	0.442	0.096	0.733	**<0.001**	0.073		**0.028**	**0.004**	**0.020**	**0.020**	0.085	0.287	0.098	0.323
*N*	73	73	75	75	75	73	75	Wrist circum-ference	103	103	102	103	103	96	97
ρ	0.304	0.742	0.311	0.207	0.088	0.493	0.211		0.422	0.494	0.433	0.449	0.514	0.244	0.268
*P*	**0.009**	**<0.001**	**0.007**	0.075	0.451	**<0.001**	0.069		**<0.001**	**<0.001**	**<0.001**	**<0.001**	**<0.001**	**0.017**	**0.008**
*N*	75	75	77	77	77	75	77	73	Waist circum-ference	107	106	107	107	100	101
ρ	0.004	0.839	0.325	0.178	0.044	0.427	0.200	0.641		0.732	0.654	0.685	0.406	0.068	0.258
*P*	0.969	**<0.001**	**0.004**	0.121	0.702	**<0.001**	0.082	**<0.001**		**<0.001**	**<0.001**	**<0.001**	**<0.001**	0.501	**0.009**
*N*	75	75	77	77	77	75	77	73	77	Hip circum-ference	106	107	107	100	101
ρ	0.031	0.878	0.397	0.199	−0.002	0.447	0.240	0.684	0.861		0.842	0.746	0.594	0.187	0.394
*P*	0.794	**<0.001**	**<0.001**	0.083	0.986	**<0.001**	**0.035**	**<0.001**	**<0.001**		**<0.001**	**<0.001**	**<0.001**	0.063	**<0.001**
*N*	77	77	79	79	79	77	79	75	77	77	Thigh circum-ference	106	106	99	100
ρ	0.184	0.821	0.309	0.055	0.061	0.397	0.144	0.632	0.614	0.728		0.754	0.546	0.224	0.454
*P*	0.109	**<0.001**	**0.006**	0.628	0.596	**<0.001**	0.207	**<0.001**	**<0.001**	**<0.001**		**<0.001**	**<0.001**	**0.026**	**<0.001**
*N*	77	77	79	79	79	77	79	75	77	77	79	Calf circum-ference	107	100	101
ρ	0.164	0.788	0.263	0.148	0.187	0.477	0.033	0.702	0.603	0.742	0.775		0.592	0.171	0.470
*P*	0.154	**<0.001**	**0.019**	0.194	0.098	**<0.001**	0.776	**<0.001**	**<0.001**	**<0.001**	**<0.001**		**<0.001**	0.088	**<0.001**
*N*	77	77	79	79	79	77	79	75	77	77	79	79	Ankle circum-ference	100	101
ρ	0.426	0.681	0.235	0.172	0.108	0.452	−0.003	0.736	0.526	0.586	0.587	0.750		0.537	0.538
*P*	**<0.001**	**<0.001**	**0.037**	0.130	0.345	**<0.001**	0.980	**<0.001**	**<0.001**	**<0.001**	**<0.001**	**<0.001**		**<0.001**	**<0.001**
*N*	74	74	76	76	76	74	76	72	75	75	76	76	76	Foot length	100
ρ	0.722	0.387	0.328	0.225	0.146	0.174	−0.001	0.469	0.217	0.223	0.249	0.388	0.582		0.405
*P*	**<0.001**	**0.001**	**0.004**	0.051	0.208	0.138	0.993	**<0.001**	0.061	0.055	**0.030**	**0.001**	**<0.001**		**<0.001**
*N*	75	75	77	77	77	75	77	73	76	76	77	77	77	77	Foot breadth
ρ	0.266	0.541	0.198	0.216	0.117	0.314	0.027	0.626	0.375	0.408	0.379	0.642	0.645	0.567	
*P*	**0.021**	**<0.001**	0.085	0.059	0.312	**0.006**	0.817	**<0.001**	**0.001**	**<0.001**	**0.001**	**<0.001**	**<0.001**	**<0.001**	

Spearman correlations. *P* < 0.05 in bold.

**Table A6:** Inter-correlations between male body measurements divided by height (alcohol-dependent in-patients bottom left, healthy control subjects top right).

*N*	Head circum-ference	133	133	133	132	128	133	133	133	132	131	105	107
ρ		0.642	0.488	0.253	0.299	0.497	0.431	0.449	0.421	0.511	0.502	0.321	0.383
*P*		**<0.001**	**<0.001**	**0.003**	**<0.001**	**<0.001**	**<0.001**	**<0.001**	**<0.001**	**<0.001**	**<0.001**	**0.001**	**<0.001**
*N*	107	Bitragion head arc	133	133	132	128	133	133	133	132	131	105	107
ρ	0.547		0.486	0.085	0.172	0.297	0.227	0.237	0.230	0.229	0.336	0.280	0.255
*P*	**<0.001**		**<0.001**	0.333	**0.049**	**0.001**	**0.009**	**0.006**	**0.008**	**0.008**	**<0.001**	**0.004**	**0.008**
*N*	107	107	Sagittal head arc	133	132	128	133	133	133	132	131	105	107
ρ	0.459	0.374		0.064	0.196	0.156	0.101	0.162	0.222	0.232	0.246	0.080	0.084
*P*	**<0.001**	**<0.001**		0.467	**0.024**	0.079	0.246	0.063	**0.010**	**0.007**	**0.005**	0.416	0.390
*N*	106	106	106	Mandibular arc	132	128	133	133	133	132	131	105	107
ρ	0.390	0.183	−0.004		0.338	0.397	0.297	0.284	0.194	0.362	0.374	0.208	0.151
*P*	**<0.001**	0.061	0.964		**<0.001**	**<0.001**	**0.001**	**0.001**	**0.025**	**<0.001**	**<0.001**	**0.033**	0.121
*N*	106	106	106	105	Lip-chin distance	127	132	132	132	131	130	104	106
ρ	0.360	0.198	−0.147	0.434		0.253	0.217	0.224	0.281	0.298	0.241	0.242	0.141
*P*	**<0.001**	**0.042**	0.132	**<0.001**		**0.004**	**0.012**	**0.010**	**0.001**	**0.001**	**0.006**	**0.013**	0.149
*N*	102	102	102	101	101	Wrist circum-ference	128	128	128	127	126	101	103
ρ	0.388	0.195	−0.039	0.449	0.378		0.602	0.644	0.510	0.590	0.601	0.271	0.404
*P*	**<0.001**	**0.050**	0.694	**<0.001**	**<0.001**		**<0.001**	**<0.001**	**<0.001**	**<0.001**	**<0.001**	**0.006**	**<0.001**
*N*	106	106	106	105	105	102	Waist circum-ference	133	133	132	131	105	107
ρ	0.350	0.198	−0.085	0.402	0.275	0.559		0.810	0.633	0.623	0.489	0.181	0.352
*P*	**<0.001**	**0.041**	0.388	**<0.001**	**0.004**	**<0.001**		**<0.001**	**<0.001**	**<0.001**	**<0.001**	0.065	**<0.001**
*N*	106	106	106	105	105	102	106	Hip circum-ference	133	132	131	105	107
ρ	0.366	0.199	−0.047	0.408	0.283	0.573	0.842		0.783	0.694	0.519	0.142	0.348
*P*	**<0.001**	**0.041**	0.631	**<0.001**	**0.003**	**<0.001**	**<0.001**		**<0.001**	**<0.001**	**<0.001**	0.149	**<0.001**
*N*	106	106	106	105	105	102	106	106	Thigh circum-ference	132	131	105	107
ρ	0.398	0.019	0.004	0.416	0.290	0.575	0.712	0.709		0.714	0.452	−0.059	0.263
*P*	**<0.001**	0.849	0.971	**<0.001**	**0.003**	**<0.001**	**<0.001**	**<0.001**		**<0.001**	**<0.001**	0.547	**0.006**
*N*	106	106	106	105	105	102	106	106	106	Calf circum-ference	131	104	106
ρ	0.330	0.158	0.076	0.322	0.182	0.598	0.625	0.586	0.695		0.533	0.088	0.439
*P*	**0.001**	0.107	0.439	**0.001**	0.062	**<0.001**	**<0.001**	**<0.001**	**<0.001**		**<0.001**	0.376	**<0.001**
*N*	106	106	106	105	105	102	106	106	106	106	Ankle circum-ference	103	105
ρ	0.434	0.260	−0.045	0.444	0.452	0.584	0.532	0.544	0.534	0.631		0.399	0.526
*P*	**<0.001**	**0.007**	0.644	**<0.001**	**<0.001**	**<0.001**	**<0.001**	**<0.001**	**<0.001**	**<0.001**		**<0.001**	**<0.001**
*N*	99	99	99	98	98	95	98	98	98	98	98	Foot length	105
ρ	0.196	0.140	0.068	0.332	0.373	0.370	0.312	0.354	0.290	0.296	0.523		0.356
*P*	0.052	0.166	0.505	**0.001**	**<0.001**	**<0.001**	**0.002**	**<0.001**	**0.004**	**0.003**	**<0.001**		**<0.001**
*N*	100	100	100	99	99	95	99	99	99	99	99	99	Foot breadth
ρ	0.411	0.282	0.040	0.271	0.292	0.538	0.351	0.403	0.373	0.472	0.629	0.436	
*P*	**<0.001**	**0.004**	0.695	**0.007**	**0.003**	**<0.001**	**<0.001**	**<0.001**	**<0.001**	**<0.001**	**<0.001**	**<0.001**	

Spearman correlations. *P* < 0.05 in bold.

**Table A7:** Inter-correlations between female body measurements divided by height (alcohol-dependent in-patients bottom left, healthy control subjects top right).

*N*	Head circum-ference	107	107	106	107	103	107	107	106	107	107	100	101
ρ		0.768	0.518	0.375	0.322	0.363	0.396	0.445	0.457	0.447	0.479	0.329	0.594
*P*		**<0.001**	**<0.001**	**<0.001**	**0.001**	**<0.001**	**<0.001**	**<0.001**	**<0.001**	**<0.001**	**<0.001**	**0.001**	**<0.001**
*N*	77	Bitragion head arc	107	106	107	103	107	107	106	107	107	100	101
ρ	0.618		0.537	0.344	0.357	0.203	0.249	0.279	0.335	0.301	0.379	0.260	0.512
*P*	**<0.001**		**<0.001**	**<0.001**	**<0.001**	**0.039**	**0.010**	**0.004**	**<0.001**	**0.002**	**<0.001**	**0.009**	**<0.001**
*N*	77	77	Sagittal head arc	106	107	103	107	107	106	107	107	100	101
ρ	0.582	0.480		0.092	0.270	−0.032	−0.027	0.155	0.195	0.101	0.094	0.148	0.303
*P*	**<0.001**	**<0.001**		0.346	**0.005**	0.746	0.782	0.111	**0.045**	0.303	0.334	0.142	**0.002**
*N*	75	75	75	Mandibular arc	106	103	106	106	105	106	106	99	100
ρ	0.444	0.413	0.207		0.402	0.361	0.509	0.591	0.543	0.513	0.559	0.267	0.491
*P*	**<0.001**	**<0.001**	0.074		**<0.001**	**<0.001**	**<0.001**	**<0.001**	**<0.001**	**<0.001**	**<0.001**	**0.008**	**<0.001**
*N*	77	77	77	75	Lip-chin distance	103	107	107	106	107	107	100	101
ρ	0.388	0.222	−0.110	0.364		0.278	0.333	0.327	0.291	0.255	0.223	0.295	0.272
*P*	**<0.001**	0.052	0.340	**0.001**		**0.005**	**<0.001**	**0.001**	**0.002**	**0.008**	**0.021**	**0.003**	**0.006**
*N*	73	73	73	71	73	Wrist circum-ference	103	103	102	103	103	96	97
ρ	0.421	0.271	0.292	0.482	0.287		0.514	0.552	0.515	0.517	0.579	0.220	0.361
*P*	**<0.001**	**0.020**	**0.012**	**<0.001**	**0.014**		**<0.001**	**<0.001**	**<0.001**	**<0.001**	**<0.001**	**0.032**	**<0.001**
*N*	75	75	75	73	75	71	Waist circum-ference	107	106	107	107	100	101
ρ	0.386	0.282	0.196	0.484	0.256	0.632		0.761	0.671	0.724	0.579	0.201	0.467
*P*	**0.001**	**0.014**	0.092	**<0.001**	**0.027**	**<0.001**		**<0.001**	**<0.001**	**<0.001**	**<0.001**	**0.045**	**<0.001**
*N*	75	75	75	73	75	71	75	Hip circum-ference	106	107	107	100	101
ρ	0.506	0.323	0.219	0.537	0.290	0.768	0.889		0.827	0.762	0.697	0.242	0.527
*P*	**<0.001**	**0.005**	0.059	**<0.001**	**0.012**	**<0.001**	**<0.001**		**<0.001**	**<0.001**	**<0.001**	**0.015**	**<0.001**
*N*	77	77	77	75	77	73	75	75	Thigh circum-ference	106	106	99	100
ρ	0.271	0.077	0.118	0.384	0.204	0.653	0.653	0.739		0.785	0.673	0.289	0.558
*P*	**0.017**	0.508	0.308	**0.001**	0.075	**<0.001**	**<0.001**	**<0.001**		**<0.001**	**<0.001**	**0.004**	**<0.001**
*N*	77	77	77	75	77	73	75	75	77	Calf circum-ference	107	100	101
ρ	0.309	0.195	0.253	0.455	0.095	0.701	0.611	0.752	0.735		0.717	0.237	0.620
*P*	**0.006**	0.090	**0.026**	**<0.001**	0.411	**<0.001**	**<0.001**	**<0.001**	**<0.001**		**<0.001**	**0.018**	**<0.001**
*N*	77	77	77	75	77	73	75	75	77	77	Ankle circum-ference	100	101
ρ	0.229	0.168	0.073	0.435	0.116	0.699	0.548	0.664	0.557	0.725		0.445	0.615
*P*	**0.045**	0.145	0.530	**<0.001**	0.315	**<0.001**	**<0.001**	**<0.001**	**<0.001**	**<0.001**		**<0.001**	**<0.001**
*N*	74	74	74	72	74	70	73	73	74	74	74	Foot length	100
ρ	0.173	0.149	0.137	0.122	0.098	0.362	0.218	0.292	0.114	0.363	0.464		0.453
*P*	0.140	0.206	0.246	0.306	0.408	**0.002**	0.064	**0.012**	0.332	**0.001**	**<0.001**		**<0.001**
*N*	75	75	75	73	75	71	74	74	75	75	75	74	Foot breadth
ρ	0.348	0.404	0.267	0.392	0.138	0.624	0.404	0.477	0.344	0.605	0.601	0.463	
*P*	**0.002**	**<0.001**	**0.021**	**0.001**	0.239	**<0.001**	**<0.001**	**<0.001**	**0.002**	**<0.001**	**<0.001**	**<0.001**	

Spearman correlations. *P* < 0.05 in bold.

**Table A8:** Inter-correlations between male body measurements residualized on height (alcohol-dependent in-patients bottom left, healthy control subjects top right).

*N*	Head circum-ference	133	133	133	132	128	133	133	133	132	131	105	107
ρ		0.459	0.384	0.125	0.246	0.390	0.275	0.232	0.228	0.333	0.379	0.336	0.226
*P*		**<0.001**	**<0.001**	0.153	**0.005**	**<0.001**	**0.001**	**0.007**	**0.008**	**<0.001**	**<0.001**	**<0.001**	**0.019**
*N*	107	Bitragion head arc	133	133	132	128	133	133	133	132	131	105	107
ρ	0.309		0.323	−0.040	0.097	0.142	0.063	0.047	0.048	0.041	0.176	0.227	0.101
*P*	**0.001**		**<0.001**	0.651	0.269	0.109	0.470	0.591	0.584	0.642	**0.044**	**0.020**	0.300
*N*	107	107	Sagittal head arc	133	132	128	133	133	133	132	131	105	107
ρ	0.239	0.251		−0.015	0.136	0.013	−0.073	−0.023	0.069	0.074	0.130	0.035	−0.041
*P*	**0.013**	**0.009**		0.864	0.120	0.881	0.403	0.794	0.432	0.401	0.138	0.725	0.673
*N*	106	106	106	Mandibular arc	132	128	133	133	133	132	131	105	107
ρ	0.307	0.086	−0.139		0.320	0.355	0.245	0.156	0.096	0.291	0.305	0.202	0.096
*P*	**0.001**	0.383	0.156		**<0.001**	**<0.001**	**0.004**	0.073	0.273	**0.001**	**<0.001**	**0.039**	0.326
*N*	106	106	106	105	Lip-chin distance	127	132	132	132	131	130	104	106
ρ	0.355	0.089	−0.278	0.420		0.209	0.158	0.136	0.204	0.248	0.181	0.205	0.104
*P*	**<0.001**	0.365	**0.004**	**<0.001**		**0.018**	0.070	0.121	**0.019**	**0.004**	**0.039**	**0.037**	0.291
*N*	102	102	102	101	101	Wrist circum-ference	128	128	128	127	126	101	103
ρ	0.404	0.112	−0.105	0.434	0.384		0.578	0.509	0.418	0.522	0.533	0.270	0.372
*P*	**<0.001**	0.262	0.295	**<0.001**	**<0.001**		**<0.001**	**<0.001**	**<0.001**	**<0.001**	**<0.001**	**0.006**	**<0.001**
*N*	106	106	106	105	105	102	Waist circum-ference	133	133	132	131	105	107
ρ	0.401	0.152	−0.141	0.398	0.273	0.549		0.732	0.551	0.572	0.399	0.179	0.298
*P*	**<0.001**	0.120	0.149	**<0.001**	**0.005**	**<0.001**		**<0.001**	**<0.001**	**<0.001**	**<0.001**	0.068	**0.002**
*N*	106	106	106	105	105	102	106	Hip circum-ference	133	132	131	105	107
ρ	0.350	0.151	−0.096	0.350	0.212	0.494	0.796		0.763	0.612	0.397	0.071	0.233
*P*	**<0.001**	0.122	0.326	**<0.001**	**0.030**	**<0.001**	**<0.001**		**<0.001**	**<0.001**	**<0.001**	0.474	**0.016**
*N*	106	106	106	105	105	102	106	106	Thigh circum-ference	132	131	105	107
ρ	0.381	−0.042	−0.089	0.370	0.264	0.522	0.715	0.709		0.666	0.362	−0.122	0.152
*P*	**<0.001**	0.671	0.365	**<0.001**	**0.007**	**<0.001**	**<0.001**	**<0.001**		**<0.001**	**<0.001**	0.216	0.117
*N*	106	106	106	105	105	102	106	106	106	Calf circum-ference	131	104	106
ρ	0.282	0.066	−0.010	0.298	0.160	0.573	0.609	0.545	0.689		0.447	0.042	0.356
*P*	**0.003**	0.502	0.920	**0.002**	0.104	**<0.001**	**<0.001**	**<0.001**	**<0.001**		**<0.001**	0.669	**<0.001**
*N*	106	106	106	105	105	102	106	106	106	106	Ankle circum-ference	103	105
ρ	0.396	0.168	−0.171	0.398	0.435	0.562	0.540	0.501	0.519	0.616		0.382	0.495
*P*	**<0.001**	0.085	0.080	**<0.001**	**<0.001**	**<0.001**	**<0.001**	**<0.001**	**<0.001**	**<0.001**		**<0.001**	**<0.001**
*N*	99	99	99	98	98	95	98	98	98	98	98	Foot length	105
ρ	0.190	0.082	0.018	0.306	0.370	0.370	0.303	0.284	0.260	0.293	0.505		0.356
*P*	0.060	0.419	0.856	**0.002**	**<0.001**	**<0.001**	**0.002**	**0.005**	**0.010**	**0.003**	**<0.001**		**<0.001**
*N*	100	100	100	99	99	95	99	99	99	99	99	99	Foot breadth
ρ	0.304	0.166	−0.085	0.221	0.257	0.519	0.367	0.341	0.317	0.448	0.584	0.428	
*P*	**0.002**	0.099	0.400	**0.028**	**0.010**	**<0.001**	**<0.001**	**0.001**	**0.001**	**<0.001**	**<0.001**	**<0.001**	

Spearman correlations. *P* < 0.05 in bold.

**Table A9:** Inter-correlations between female body measurements residualized on height (alcohol-dependent in-patients bottom left, healthy control subjects top right).

*N*	Head circum-ference	107	107	106	107	103	107	107	106	107	107	100	101
ρ		0.652	0.372	0.219	0.265	0.231	0.267	0.290	0.301	0.245	0.296	0.210	0.354
*P*		**<0.001**	**<0.001**	**0.024**	**0.006**	**0.019**	**0.005**	**0.002**	**0.002**	**0.011**	**0.002**	**0.036**	**<0.001**
*N*	77	Bitragion head arc	107	106	107	103	107	107	106	107	107	100	101
ρ	0.380		0.388	0.205	0.283	0.060	0.095	0.023	0.102	0.064	0.198	0.165	0.324
*P*	**0.001**		**<0.001**	**0.035**	**0.003**	0.549	0.332	0.811	0.296	0.515	**0.041**	0.101	**0.001**
*N*	77	77	Sagittal head arc	106	107	103	107	107	106	107	107	100	101
ρ	0.351	0.303		−0.056	0.187	−0.170	−0.148	0.000	0.067	−0.057	−0.075	0.061	0.111
*P*	**0.002**	**0.007**		0.567	0.054	0.086	0.128	0.996	0.493	0.563	0.441	0.548	0.269
*N*	75	75	75	Mandibular arc	106	103	106	106	105	106	106	99	100
ρ	0.294	0.268	0.049		0.350	0.334	0.478	0.469	0.443	0.438	0.497	0.236	0.404
*P*	**0.011**	**0.020**	0.676		**<0.001**	**0.001**	**<0.001**	**<0.001**	**<0.001**	**<0.001**	**<0.001**	**0.019**	**<0.001**
*N*	77	77	77	75	Lip-chin distance	103	107	107	106	107	107	100	101
ρ	0.279	0.054	−0.339	0.291		0.255	0.299	0.203	0.213	0.170	0.145	0.279	0.190
*P*	**0.014**	0.643	**0.003**	**0.011**		**0.009**	**0.002**	**0.036**	**0.028**	0.080	0.137	**0.005**	0.057
*N*	73	73	73	71	73	Wrist circum-ference	103	103	102	103	103	96	97
ρ	0.291	0.120	0.180	0.429	0.258		0.499	0.458	0.401	0.452	0.521	0.217	0.324
*P*	**0.012**	0.310	0.128	**<0.001**	**0.028**		**<0.001**	**<0.001**	**<0.001**	**<0.001**	**<0.001**	**0.033**	**0.001**
*N*	75	75	75	73	75	71	Waist circum-ference	107	106	107	107	100	101
ρ	0.304	0.163	0.095	0.417	0.213	0.620		0.695	0.622	0.684	0.507	0.201	0.413
*P*	**0.008**	0.162	0.418	**<0.001**	0.066	**<0.001**		**<0.001**	**<0.001**	**<0.001**	**<0.001**	**0.045**	**<0.001**
*N*	75	75	75	73	75	71	75	Hip circum-ference	106	107	107	100	101
ρ	0.382	0.207	0.035	0.446	0.210	0.671	0.863		0.838	0.722	0.589	0.135	0.354
*P*	**0.001**	0.074	0.767	**<0.001**	0.071	**<0.001**	**<0.001**		**<0.001**	**<0.001**	**<0.001**	0.181	**<0.001**
*N*	77	77	77	75	77	73	75	75	Thigh circum-ference	106	106	99	100
ρ	0.207	−0.023	0.030	0.296	0.099	0.541	0.590	0.727		0.746	0.562	0.193	0.410
*P*	0.071	0.844	0.796	**0.010**	0.392	**<0.001**	**<0.001**	**<0.001**		**<0.001**	**<0.001**	0.055	**<0.001**
*N*	77	77	77	75	77	73	75	75	77	Calf circum-ference	107	100	101
ρ	0.214	0.110	0.188	0.413	0.032	0.655	0.585	0.747	0.740		0.643	0.174	0.509
*P*	0.062	0.342	0.101	**<0.001**	0.784	**<0.001**	**<0.001**	**<0.001**	**<0.001**		**<0.001**	0.083	**<0.001**
*N*	77	77	77	75	77	73	75	75	77	77	Ankle circum-ference	100	101
ρ	0.157	0.115	0.058	0.388	0.032	0.639	0.511	0.634	0.542	0.725		0.432	0.533
*P*	0.172	0.318	0.615	**0.001**	0.783	**<0.001**	**<0.001**	**<0.001**	**<0.001**	**<0.001**		**<0.001**	**<0.001**
*N*	74	74	74	72	74	70	73	73	74	74	74	Foot length	100
ρ	0.212	0.180	0.176	0.130	0.112	0.403	0.247	0.294	0.098	0.369	0.474		0.453
*P*	0.070	0.125	0.134	0.275	0.342	**0.001**	**0.035**	**0.012**	0.409	**0.001**	**<0.001**		**<0.001**
*N*	75	75	75	73	75	71	74	74	75	75	75	74	Foot breadth
ρ	0.153	0.240	0.132	0.331	0.088	0.585	0.371	0.378	0.263	0.559	0.569	0.506	
*P*	0.190	**0.038**	0.258	**0.004**	0.452	**<0.001**	**0.001**	**0.001**	**0.022**	**<0.001**	**<0.001**	**<0.001**	

Spearman correlations. *P* < 0.05 in bold.
